# Part 1 - imidazolines and the changing face of nasal decongestants

**DOI:** 10.3389/fphar.2025.1655252

**Published:** 2025-11-28

**Authors:** Rebecca J. Stinson, Laura R. Sadofsky

**Affiliations:** Biomedical Institute for Multimorbidity, Centre for Biomedicine, Hull York Medical School, University of Hull, Hull, United Kingdom

**Keywords:** nasal decongestants, imidazolines, oxymetazoline, sympathomimetic, nasal decongestants/adverse effects, imidazoline receptor, xylometazoline

## Abstract

Imidazolines are sympathomimetic drugs used to treat a range of conditions including nasal congestion, ocular disorders, and hypertension. Imidazolines were discovered over 150 years ago. However, it was research from the 1940s onwards which established the therapeutic benefits of imidazolines. Although there is extensive literature describing imidazolines, the history and timeline of their development is not well documented. This review focuses on the evolution of imidazoline pharmacology particularly those used in nasal decongestants, naphazoline, tetrahydrozoline, xylometazoline and oxymetazoline. These derivatives activate the α_1_-and α_2_-adrenergic receptors with varying degrees of selectivity, to provide decongestive relief through vasoconstriction. This reduces swelling of the nasal mucosa, delivering both subjective and objective relief from congestion. Each new imidazoline derivative has improved onset and duration of action, resulting in treatments with enhanced efficacy, tolerability, and safety. Although these advancements allow for less frequent dosing with comparable effects, the importance of correct usage for optimal benefit cannot be overstated. These nasal decongestants are considered safe when used as recommended however, rhinitis medicamentosa, characterized by chronic nasal congestion, can occur with excessive use. Imidazolines are an important class of compounds which have shown improvements in efficacy and safety over time. However, further improvements could be made with more advances in understanding their pharmacology.

## 1 Introduction

**FIGURE 1:**
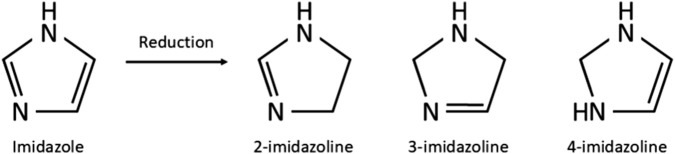
Chemical structure of imidazole and the imidazolines produced through reduction of imidazole.

Imidazolines are a valuable class of bioactive compounds, forming the active ingredients in numerous formulations of topical nasal decongestant sprays, topical ocular treatments and antihypertensives to name but a few (Krasavin, 2015; Mehedi and Tepe, 2020; National Center for Biotechnology Information, 2024). Their success is due to the relatively high efficacy and tolerability, coupled with the low risk of serious side effects experienced when taken correctly, making them a popular over-the-counter (OTC) and prescription treatment for a wide range of aliments (Trinh et al., 2024; Black and Remsen, 1980; Wang et al., 2024). Over 50 different imidazolines have been identified, whilst many of these have been synthesised for their pharmacological properties, several are also naturally occurring, taking the form of biological compounds such as purines and histidine (Tyagi et al., 2007). The uses of imidazolines are far reaching, being bioactive in both their synthetic and natural forms. Therapeutic uses of imidazolines include treatment of fungal diseases, hypertension, parasitic worm infections, allergies, inflammation, pain, hyperglycaemia, and cancer, they also have potential for treating Alzheimer’s and Parkinson’s disease (Mehedi and Tepe, 2020; Kabi et al., 2024). Beyond pharmacological applications, imidazolines are utilised in immobilised metal affinity chromatography, prevention of copper corrosion, photography, electronics and as a fire retardant (Bhatnagar et al., 2011). Three isomers of imidazolines exist, known as 2-, 3-, and 4-imidazoline, which reflects the position of the double bond within the ring structure (Figure 1). Of these, 2-imidazoline is of the greatest importance owing to its numerous biologically active compounds and the commercial benefit they provide in pharmacology and beyond (Krasavin, 2015; Liu and Du, 2009).

**FIGURE 2:**
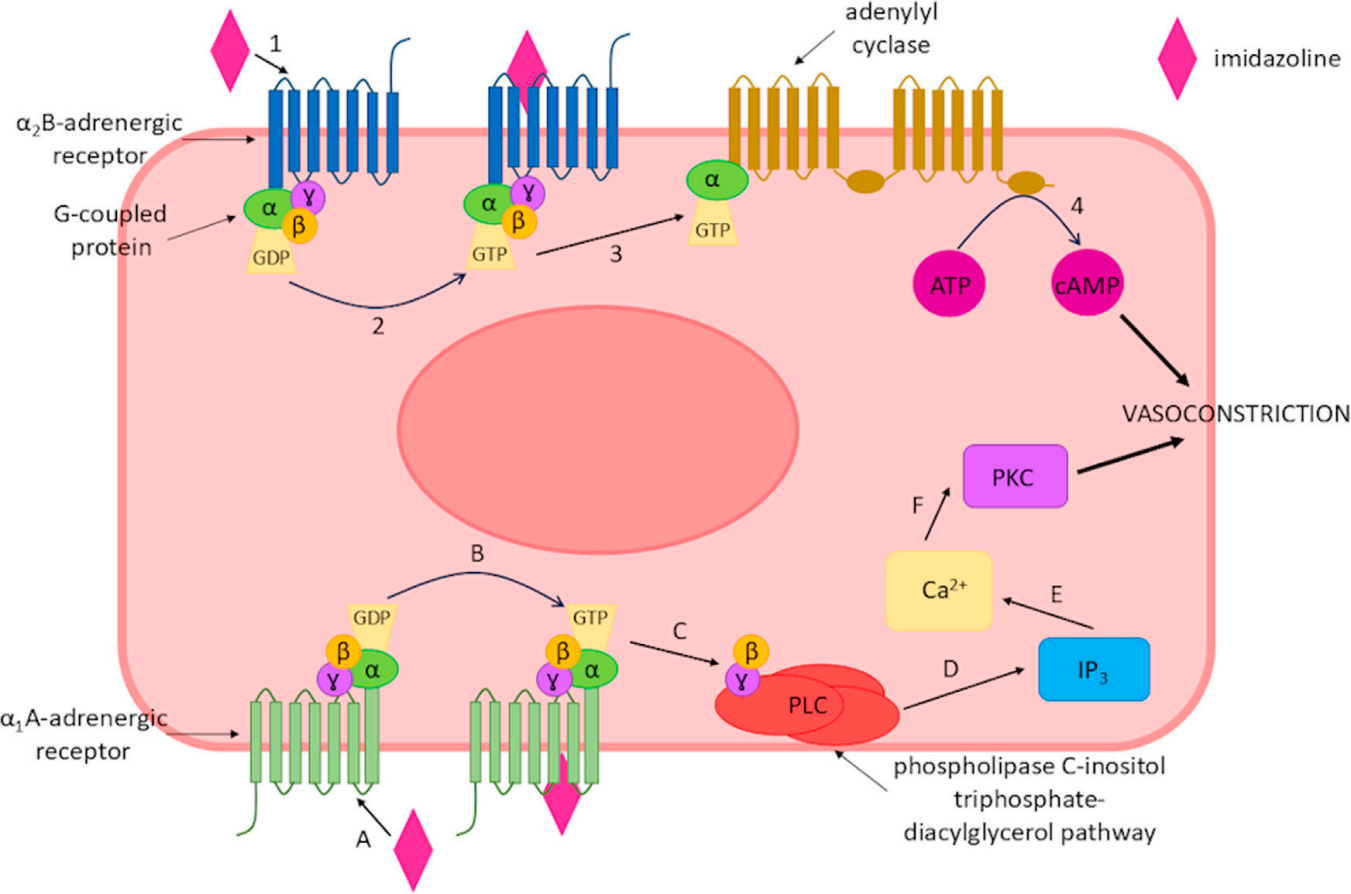
Imidazoline-triggered activation of α-adrenergic receptors leading to vasoconstriction of smooth muscle. Binding of an imidazoline derivative to the α1A-adrenergic receptor. **(A)** activates the receptor **(B)**, this subsequently activates the phospholipase C-inositol triphosphate-diacylglycerol pathway **(C,D)**, which increases intracellular calcium **(E)** and activates protein kinase C **(F)** leading to vasoconstriction of the smooth muscle. Alternatively, an imidazoline derivate binds to α2B-adrenergic receptor (1), resulting in receptor activation (2) which subsequently increases adenylyl cyclase (3) and cAMP production (4) leading to vasoconstriction.

Imidazolines are classified as sympathomimetic drugs and are frequently used as nasal decongestants alongside sympathomimetic amines such as phenylephrine, as they have the benefit of being lipophilic and rapidly absorbed (Rizvic et al., 2017; Wenzel et al., 2004). Sympathomimetic drugs activate the sympathetic nervous system either directly or indirectly, as they mimic endogenous molecule action or intracellular signalling pathways (Costa et al., 2022). This creates an effect through the activation of α_1_-and α_2_-adrenergic receptors, a class of G protein-coupled receptors (GPCRs) with varying degrees of selectivity. Those that work indirectly increase the level of catecholamine in the synaptic cleft, which activates the adrenergic receptors in the same manner as directly acting sympathomimetics (Costa et al., 2022; Hieble and Ruffolo, 1992; Xu et al., 2022). Upon activation by an imidazoline the response of the adrenergic receptor depends on its type of receptor. α_1_-adrenergic receptors couple with G proteins activating the phospholipase C-inositol triphosphate-diacylglycerol pathway, which subsequently releases intracellular calcium and activates protein kinase C. Whilst, α_2_-adrenergic receptors may also interact with stimulatory G proteins and increase both adenylyl cyclase and cAMP production (Maaliki et al., 2024) (Figure 2).

These variations between receptor activation may explain why the different imidazoline derivatives produce differing responses, with the aforementioned responses resulting in the vasoconstriction effect (Maaliki et al., 2024). Whilst imidazolines are sympathomimetic drugs, their mechanism of action cannot fully be explained by their interaction with adrenergic receptors alone, thus specific imidazoline receptors have been proposed and identified which although not fully elucidated has led to the development of improved imidazoline derivatives (Bousquet, 1995). The imidazoline compounds which are now available for the treatment of numerous aliments is the culmination of nearly 150 years of research. However, whilst the literature relating to imidazolines is extensive, there is a limited collective overview of these derivatives. Thus, the story of how and why we see the development from early derivates to those in current widespread use is less clear. Here, we aim to review the history of this class of compounds and in doing so provide a timeline of development of the different imidazoline derivatives, particularly those which feature in topical nasal decongestants. The drivers behind the search for new derivates and the role receptors have played in the research and understanding of this important class of compounds will be considered.

## 2 Imidazolines: a brief past to present

The imidazoline precursor, imidazole, a hetercyclic organic compound, was first produced as early as the 1850s (Bhatnagar et al., 2011). Imidazolines are produced by reduction of imidazoles and were first synthesised by a German chemist in 1888 (Mehedi and Tepe, 2020) (Figure 1). After the late 1880s there appears to be no further development of this class of drugs, with no references in the literature being evident until the late 1930s with the synthesis of tolazoline and shortly after naphazoline hydrochloride in the early 1940s (National Center for Biotechnology Information, 2024; Szabo, 2002). Since their initial discovery, imidazolines have been extensively developed. The earliest examples were used to treat nasal congestion and shortly after eye irritation however, early cases of acute toxicity with naphazoline resulted in subsequent developments to improve efficacy and safety (Mertins, 1947; Neistadt, 1955; Von Nordheim, 1955). Thus within 30 years the number of imidazoline derivates available for use as a nasal decongestant had risen from one to eight (Table 1).

**TABLE 1:** Imidazolines used predominantly in the treatment of nasal congestion.

Imidazoline derivative	Chemical structure and formula	Molecular weight (g/mol)	Application	WHO ATC classification^*^	Currently used in nasal decongestants
Naphazoline	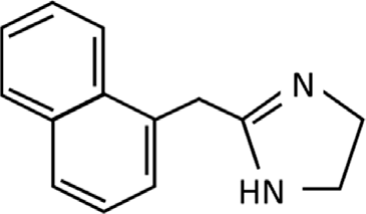 C_14_H_14_N_2_	210.28	Reduction of red eye Nasal decongestion	R01AA08 Identified as sympathomimetic, plain, DDD 0.4 mg daily	No longer widely utilised in nasal spray preparations Available in India, Brazil and eastern europe
Antazoline	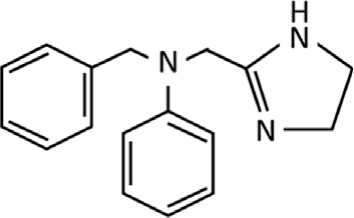 C_17_H_19_N_3_	265.36	Nasal decongestion Conjunctivitis treatment	Not listed	Not widely utilised in nasal spray preparations Can be used in conjunction with naphazoline and tetrahydrozoline Available in eastern europe
Tymazoline	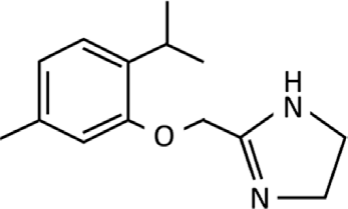 C_14_H_20_N_2_O	232.33	Nasal decongestion, specifically for rhinitis	R01AA13 Identified as sympathomimetic, plain, no daily dose	Not widely utilised in nasal spray preparations Available in a few european countries and Russia
Xylometazoline	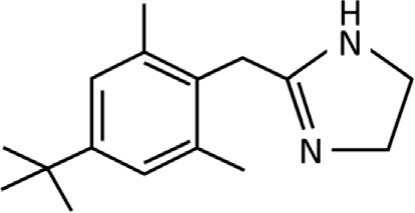 C_16_H_24_N_2_	244.38	Nasal decongestion Treatment of allergic rhinitis and sinusitis	R01AA07 Identified as sympathomimetic, plain, DDD 0.8 mg daily	Widely used in nasal spray preparation worldwide
Tetrahydrozoline	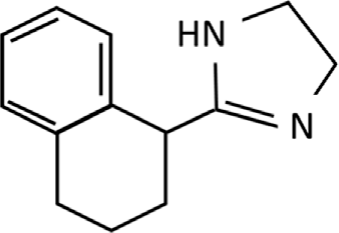 C_13_H_16_N_2_	200.29	Reduction of eye irritation Nasal/nasopharyngeal decongestion	R01AA06 Identified as sympathomimetic, plain, DDD 0.8 mg daily	No longer widely utilised in nasal spray preparations Available in US.
Oxymetazoline	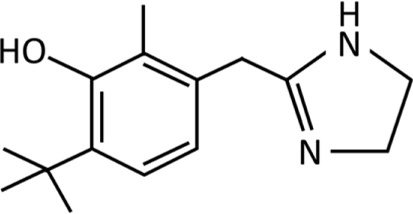 C_16_H_24_N_2_O	260.38	Rosacea treatment Reduction of eye irritation Nasal decongestion Treatment of nosebleeds Blepharoptosis treatment	R01AA05 Identified as sympathomimetic, plain, DDD 0.4 mg daily	Widely used in nasal spray preparation worldwide
Tramazoline	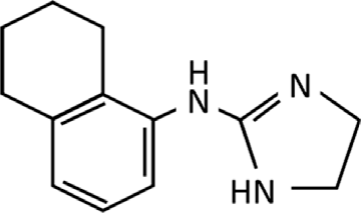 C_13_H_17_N_3_	215.30	Nasal decongestion	R01AA09 Identified as sympathomimetic, plain, no daily dose	Occasionally used in nasal spray preparations Available in some european countries, Australia and Russia
Fenoxazoline	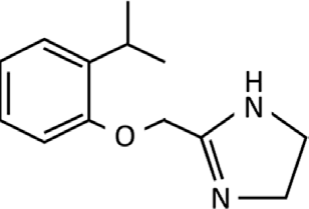 C_13_H_18_N_2_O	218.30	Nasal decongestion	R01AA12 Identified as sympathomimetic, plain, no daily dose	Not widely utilised in nasal spray preparations Available in a few european countries

*Details based on the World Health Organisation’s utilisation of the Anatomical Therapeutics Classification and defined daily dose (DDD), the average daily maintenance dose for adults may not correspond to the recommended prescribed daily dose (Wang et al., 2024; Bucaretchi et al., 2003; Bende and Pipkorn, 1987; Wishart et al., 2018; Menger, 1959; Nagane et al., 2011; Duzman et al., 1986; Shanler and Ondo, 2007; WHO Collaborating Centre for Drug Statistics Methodology, 2024; Norwegian Institute of Public Health, 2024; World health Organisation, 2024).

**FIGURE 3:**
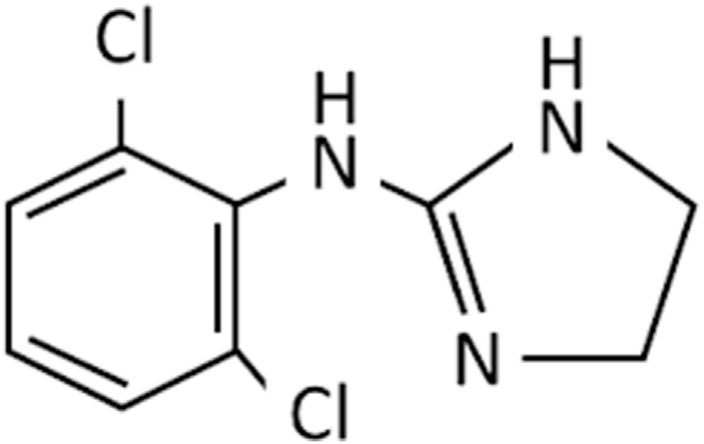
Chemical structure of clonidine.

Nasal decongestants form only part of the story of the imidazolines. As strides were being made to improve the efficacy and safety of nasal decongestants, alternative uses were being identified. Perhaps the most significant was the discovery of clonidine in the 1960s (Figure 3). Initially tested as a potential nasal decongestant, the significant systemic side effects it produced subsequently showed it had high efficacy as an anti-hypertensive treatment, thus switching the focus of its use (Gold et al., 2024; Stähle, 2000; van Zwieten, 1980). Furthermore, it became fundamental in developing an understanding of the interactions of imidazoline derivatives with their receptors from the 1970s onwards (Bousquet, 1995; Gold et al., 2024; Stähle, 2000). This resulted in the elucidation of the α-adrenergic receptors subtypes, α_1_ and α_2_, and later their subdivisions α_1_A, α_1_B, α_1_C, α_2_A, α_2_B, and α_2_C. Identification of these receptor subtypes enabled numerous imidazoline derivatives to be synthesised and tested based on their binding affinities (Bousquet et al., 2020; Langer, 1999; Bylund, 1985; Khan et al., 1999). However, the central action of clonidine’s hypotensive effect could not be fully explained by its interaction with α_2_-adrenergic receptors alone. Furthermore, there was no relationship between affinity towards an α-adrenergic and the extent to which an imidazoline reduced blood pressure. Suggesting that another receptor specific to imidazolines may be involved (Bousquet, 1995).

Imidazoline specific receptors were first confirmed in 1987, with three subtypes, I_1_, I_2_ and I_3_ isolated based on distinct binding and functional pharmacology, which indicated the mode of action of imidazolines is also via a pathway independent of α_2_-adrenergic receptors (Bousquet et al., 2020; Khan et al., 1999; Nutt et al., 1995). Imidazoline receptors are found throughout the body of several mammalian species including humans, predominately these are located within the brain and central nervous system however, the sites depend on the specific receptor (Bousquet et al., 2020; Khan et al., 1999; Dardonville and Rozas, 2004; Ernsberger et al., 1995; Head and Mayorov, 2006; Regunathan and Reis, 1996) (Table 2). It is worth noting that none have currently been identified specifically in the nasal passages, thus although imidazoline based nasal decongestants have been shown to interact with the α-adrenergic receptors, the role imidazoline receptors play in their mechanisms of action, if any, are yet to be established (Haenisch et al., 2010; Horie et al., 1995).

**TABLE 2:** Identified locations of imidazoline receptors in mammals including humans (Bousquet et al., 2020; Khan et al., 1999; Dardonville and Rozas, 2004; Ernsberger et al., 1995; Head and Mayorov, 2006; Regunathan and Reis, 1996).

Site of receptor	I_1_ receptor	I_2_ receptor	I_3_ receptor
Brain	✓	✓	-
Central nervous system	✓	✓	-
Kidney	✓	✓	-
Pancreas	-	✓	✓
Adrenal gland	✓	✓	-
Stomach	✓	-	-
Adipose tissue	✓	-	-
Heart	✓	✓	-
Placenta	-	✓	-
Colon	-	✓	-
Vascular smooth muscle	-	✓	-
Liver	-	✓	-
Urethra	-	✓	-
Carotid body	✓	✓	-
Prostate	✓	✓	-
Platelets	✓	-	-

**FIGURE 4:**
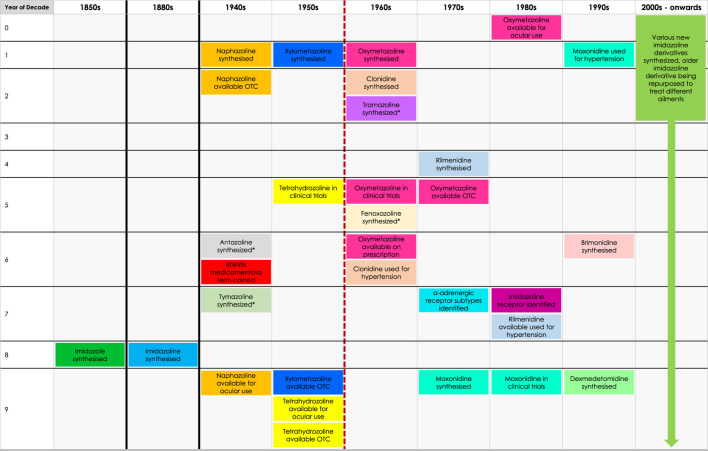
Timeline of discovery of imidazoline derivatives. Red dashed line represents the point where there is a gradual shift from early-generation to new-generation imidazoline derivatives, which can be defined as such: early-generation imidazoline derivatives were created prior to 1960, typically have higher risk of side effects, longer subjective onset of action, are shorter acting and require higher or more frequent doses. New-generation imidazoline derivatives were created after 1960, typically have fewer side effects, greater tolerability, a subjective faster onset of action, are longer acting, requiring lower or less frequent doses, and have greater α-adrenergic receptor affinity and potency. *Represents imidazoline derivatives which are less widely used and so more are difficult to define in such terms.

Whilst the role these receptors play in nasal decongestion is unclear, the identification of the I_1_ receptor has enabled the development of new imidazoline derivates, that are better tolerated than earlier derivatives (Figure 4). These newer derivatives tend to have a higher affinity and potency for α_2_-adrenergic receptors, additionally they also have some affinity to α_1_-adrenergic receptors. Whilst, the impact of these differences has not been fully elucidated, it may go some way to explain the faster onset of action and different dosages compared to earlier imidazolines (Wang et al., 2024; Khan et al., 1999; Haenisch et al., 2010; Jones, 2021; Ruffolo and Waddell, 1982; Sanders et al., 1975). Furthermore, they benefit from having a higher affinity to imidazoline receptors compared to α_2_-adrenergic receptors (Bousquet et al., 2020; Head and Mayorov, 2006; Erszegi et al., 2024; Musgrav et al., 1998; Muramatsu and Kigoshi, 1992; Piletz et al., 2000). These newer generation imidazolines have also been shown to have a longer lasting effect. The higher efficacy means patients require fewer doses per day to achieve the desired reduction in symptoms, making them a safer and more tolerable alternative to the earlier generation imidazolines (Schafer et al., 1992; Druce et al., 2018; Reinecke and Tschaikin, 2005). This successful characterisation of the imidazoline binding sites not only helped improved the patient experience in regards to nasal decongestion, but has also seen research diversify beyond the management of hypertension and congestion to fields including pain, epilepsy, inflammation, cancer, appetite, cell proliferation and adhesion, opioid addiction and neuroprotection (Head and Mayorov, 2006). Although the pharmacological benefit of imidazolines is wide reaching, this paper will focus on the changing landscape of imidazolines as nasal decongestants, influences on the development of new derivatives and how this has improved patient experience.

## 3 The changing landscape of imidazolines as nasal decongestants

### 3.1 History of discovery

**FIGURE 5:**
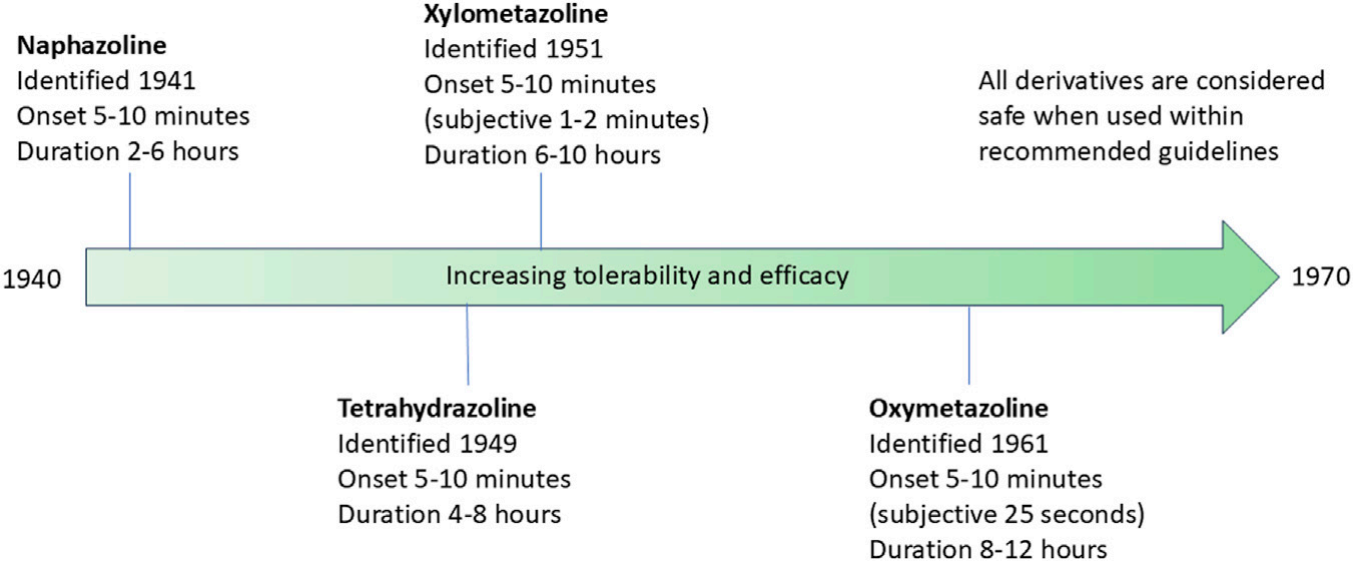
Timeline of the identification of the four most widely studied imidazoline nasal decongestants. The timeline indicates the date of identification, the onset of action and duration of action. The tolerability and efficacy of the nasal decongestions improves with each subsequent derivative.

Imidazoline derivatives form the active ingredient in a wide range of nasal decongestants which are available OTC. However, the number of derivatives which can be used in these products has not remained static, instead over a period of approximately 20 years the number continued to increase, driven by a desire to improve tolerability and efficacy, thus reducing the need for frequent dosing. The history of the four most widely studied imidazoline nasal decongestants is outlined henceforth and overviewed in Figure 5.

#### 3.1.1 Naphazoline

Naphazoline hydrochloride was first identified as an effective treatment for nasal congestion in 1941. The preparation was marketed as a nasal vasoconstrictor which functioned to reduce swelling of the nasal mucosa and was hailed at the time as non-toxic (Putnam and Herwick, 1946). Whilst, the onset of action is as little as 5 minutes, the relatively short window of activity of around 2–6 h, means naphazoline needs to be readministered relatively frequently which can result in acute toxicity, especially if delivered incorrectly leading to accidental ingestion (Wang et al., 2024; Wenzel et al., 2004). The safety implications of incorrect application are discussed below in more detail.

#### 3.1.2 Tetrahydrozoline

The need for drugs with greater efficacy, lower effective dose and a longer therapeutic window led to the production of another imidazoline, tetrahydrozoline hydrochloride, which following 4 years of clinical trials became available in 1959 (Neistadt, 1955; Menshawey and Menshawey, 2024). As with naphazoline, tetrahydrozoline also acts as a vasoconstrictor on the nasal mucosa and has an onset of action within 5–10 min of application. However, it provides the added benefit of having a prolonged effect, lasting between 4–8 h, thus requiring less frequent administration and has lower toxicity (Neistadt, 1955; Hutcheon et al., 1955; Bucaretchi et al., 2003).

#### 3.1.3 Xylometazoline

By the end of the 1940s, almost simultaneously with tetrahydrozoline, xylometazoline was developed to improve the dose and tolerability of nasal decongestants, with clinical trials beginning around 1951 (Graf et al., 2018; Steinberg, 1953). As with naphazoline and tetrahydrozoline, it acts as a vasoconstrictor on the nasal submucosa, reducing congestion caused by sinusitis and rhinitis. Although still showing some of the features of the earlier generation imidazolines, xylometazoline became the forerunner in the shift towards a newer generation of imidazolines, with improved efficacy and duration of action. As with previously described nasal decongestants, it provides rapid onset of symptom relief within 5–10 min of application however, it gives an extended period of symptom relief of between 6–10 h and may provide relief for up to 12 h (Graf et al., 2018; Steinberg, 1953; Eccles et al., 2008; Eccles et al., 2010).

#### 3.1.4 Oxymetazoline

The development of nasal vasoconstrictors did not stop at this point, instead improvements continued to be made with the synthesis of oxymetazoline. This continued development arose due to a need to improve efficacy of a nasal decongestant without causing significant reactive hyperaemia. Whilst the early-generation imidazoline derivatives proved to be longer lasting than their ephedrine and epinephrine derivative counterparts, the length of time they provided therapeutic relief from congestion was still shorter than desired for some applications (Hotovy et al., 1961). Thus, a minor modification to the nasal decongestant xylometazoline led to the development of oxymetazoline by Merck in the 1960s, producing a new-generation imidazoline which further improved efficacy and lowered toxicity (Evaluation of a nasal decongestant, 1965; Chatelet, 1965; Cartabuke et al., 2021; Hochban et al., 1999). As a result of modification, oxymetazoline can provide significant subjective and objective relief from nasal congestion for up to 12 h. Subjective relief can be experienced as quickly as 25 s after application, whilst objective relief occurs within 5–10 min (Druce et al., 2018; Reinecke and Tschaikin, 2005; Åkerland et al., 1989; Connell and Linzmayer, 1987).

#### 3.1.5 Other derivatives as nasal decongestants

Other imidazolines have been identified as having nasal decongestant properties (Table 1), although these are less widely used for this purpose than those previously described. Antazoline, synthesised in 1946 is an imidazoline and first-generation antihistamine, that is used in nasal decongestants often alongside other imidazolines, for the treatment of allergic rhinitis and hay fever (Bende and Pipkorn, 1987; Boora, 2007). In addition, it is used to treat eye and skin irritation due to allergies, has potential anti-viral properties and may help in the treatment of arrhythmia (Bende and Pipkorn, 1987; Boora, 2007; Li et al., 2021; Springer et al., 2024). Similarly, the imidazoline derivative tramazoline, synthesised in 1962, functions as a nasal decongestant with a similar efficacy to xylometazoline but lacks the wider range of uses seen in other imidazolines (Hochban et al., 1999; Maryadele et al., 2007). Synthesised in 1947, tymazoline has nasal vasoconstrictor properties which are effective against rhinitis symptoms. However, it is not widely utilised and other uses have not been determined (Maryadele et al., 2007; Wishart et al., 2018). Fenoxazoline is the final imidazoline derivative that is used as a nasal decongestant for rhinitis. Synthesised in 1965, it functions in the same manner as other derivatives although is not as widely available (Bucaretchi et al., 2003; Maryadele et al., 2007).

### 3.2 Alternative uses

Interestingly the vasoconstrictive properties of the aforementioned imidazoline derivatives have uses beyond nasal decongestion, with the most notable use being in ophthalmic applications (Tables 1, 3). The mode of action as a vasoconstrictor, initially enabled naphazoline to be utilised in topical ocular applications to provide a reduction in the red eye and swelling associated with allergic conjunctivitis (Abelson et al., 1984; Shellans et al., 1989). Similarly, tetrahydrozoline has also been shown to be an effective ocular decongestant against allergic conjunctivitis and associated red-eye (Menger, 1959). Naphazoline can also be used for the treatment of glaucoma, and more recently has shown potential for the treatment of ocular myasthenia gravis, where it may improve the tone of the muscles controlling the eyelids, reducing the extent of drooping experienced by some patients (Nagane et al., 2011). Oxymetazoline provides some overlap with naphazoline being effective in treating eye irritation due to environmental or allergic conjunctivitis or at higher concentrations to treat blepharoptosis (Duzman et al., 1986; Newland et al., 2025; Samson et al., 1980). Oxymetazoline has also been used successfully to treat epistaxis through nasal application on absorbent swabs (Katz et al., 1990; Womack et al., 2018), and topical application to the skin has been shown to reduce facial flushing and erythema associated with some types of rosacea (Shanler and Ondo, 2007).

**TABLE 3:** Described variations in duration and onset of action in the most commonly used imidazoline based nasal decongestants. Subjective onset of action is given where these are clearly discussed in literature.

Imidazoline derivative		Naphazoline	Tetrahydrozoline	Xylometazoline	Oxymetazoline
Onset of action	Objective (minutes)	5–10	5–10	5–10	5–10
	Subjective	-	-	1–2 min	25 s
Duration of action (hours)		2–6	4–8	6–10 (up to 12*)	8–12
Additional properties					
Anti-inflammatory		X	X	X	✓
Antiviral		X	X	X	✓
Antioxidant		?	X	✓	✓
Alternative treatment uses					
Allergic conjunctivitis		✓	✓	✓	✓
Gluacoma		✓	-	-	-
Ocular myasthenia gravis		✓	-	-	✓
Reduction of eye irritation		✓	✓	✓	✓
Reduction of red eye		✓	✓	✓	✓
Rosacea		-	-	-	✓
Nosebleeds		-	-	-	✓

*12-h duration recognised as a potential trend in literature, significance only noted until 10 h post application. ? naphazoline recognised as having potential antioxidant properties however, further investigation is required (Wang et al., 2024; Neistadt, 1955; Druce et al., 2018; Reinecke and Tschaikin, 2005; Bucaretchi et al., 2003; Graf et al., 2018; Steinberg, 1953; Eccles et al., 2008; Eccles et al., 2010; Abelson et al., 1984; Shellans et al., 1989; Menger, 1959; Nagane et al., 2011; Duzman et al., 1986; Newland et al., 2025; Samson et al., 1980; Katz et al., 1990; Womack et al., 2018; Shanler and Ondo, 2007).

The pharmacodynamics of the imidazoline derivatives extend beyond those relating to nasal decongestion and ophthalmology and their ability to cause vasoconstriction. Given the various aetiologies of nasal congestion, the identification of other potentially useful properties, including acting as an antioxidant, anti-inflammatory and antiviral, may aid in treating the underlying causative agents and in doing so help reduce the burden of symptoms. Naphazoline has shown potential as a possible antioxidant against radiation. Interestingly, the vasoconstrictive property of naphazoline may be partly responsible for its antioxidant effect alongside its radical scavenging ability however, the mechanisms involved are currently not fully understood (Prouillac et al., 2006; Musa and Eriksson, 2007; Ponomarev et al., 2023). Similarly, xylometazoline and oxymetazoline have also been shown to have antioxidant properties. Although the mechanisms have not been fully elucidated, this action may involve inhibition of nitric oxide synthase in the nasal passages, with oxymetazoline being rated as the more effective of the two (Westerveld et al., 1995; Westerveld et al., 2000). In addition, oxymetazoline has been shown to have potential antiviral and anti-inflammatory properties. These anti-inflammatory properties may be due to an immunomodulating effect created through inhibition of proinflammatory cytokines and lower stimulatory capacity of T-cells. Interestingly, topical application of oxymetazoline has also been shown to reduce the duration of rhinitis symptoms by 2 days when compared to saline alone (Table 3) (Reinecke and Tschaikin, 2005; Westerveld et al., 1995; Koelsch et al., 2007; Jagade et al., 2008). These additional properties may explain why some imidazoline derivatives are more effective nasal decongestants than others, with the different properties working synergistically beyond the nasal mucosa however, further studies are required to better understand and elucidate the mechanisms which are at play.

### 3.3 Comparative efficacy

The historical development of the imidazoline derivatives utilised in nasal decongestants represents a desire to improve their efficacy, potency and effectiveness (Greenstein, 1955; Wahid and Shermetaro, 2023). Whilst the objective onset of action of each new derivative has remained unchanged at around 5–10 min post application, there has been a noticeable shift towards a more rapid subjective onset of action with the more recent derivatives (Table 3) (Reinecke and Tschaikin, 2005; Eccles et al., 2008; Hochban et al., 1999). Measurements of the objective onset of action of nasal decongestants can be assessed via nasal resistance and conductance. Methods including nasal peak flow, rhinomanometry, rhinostereometry and acoustic rhinometry are widely utilised to make assessments on nasal decongestant effectiveness (Ottaviano and Fokkens, 2016). For example, a small trial with 30 healthy volunteers was performed to objectively compare the decongestive effects of oxymetazoline and xylometazoline. Using active anterior rhinomanometry and acoustic rhinometry measurements both xylometazoline and oxymetazoline were shown to have similar decongestive effects which occurred rapidly, within minutes of application and with similar duration (Eskiizmir et al., 2011). In addition measurements can be confirmed via computed tomography or magnetic resonance imaging (MRI) (Ottaviano and Fokkens, 2016). However, it is worth noting methods such as rhinomanometry and rhinostereometry can pose technical challenges which have the potential to impact the accuracy of the measurements taken. Thus, using a combination of clinical measurements and imaging is more likely to give a clearer indication of the effectiveness of a treatment (Patil and Jain, 2024; Hagen et al., 2025). As such, the effectiveness of oxymetazoline to reduce the extent of swelling in the nasal turbinate, has been confirmed through MRI and nasal patency measurements (Druce et al., 2018; Kishore et al., 2015; Pritchard et al., 2014).

Although the objective times for the onset of action can be derived via clinical measurements, giving a definitive time point for when symptom relief occurs, subjective relief is also worth considering (Eccles et al., 2008). Whilst there is limited evidence relating to the subjective relief provided from naphazoline or tertrahydrozoline application, more evidence is present for xylometazoline and oxymetazoline application. For some individuals, subjective relief from symptoms can be experience around 2 min after application of xylometazoline and as quickly as 25 s after application of oxymetazoline (Druce et al., 2018; Reinecke and Tschaikin, 2005; Eccles et al., 2008; Åkerland et al., 1989; Connell and Linzmayer, 1987). The importance of this subjective effect should not be underestimated, as any perceived improvement in symptoms is likely to positively impact patient wellbeing, as much as any objective change would. Additionally, this may also influence how and when individuals utilise nasal decongestants in order to experience maximum relief of symptoms (Eccles et al., 2008).

The duration of symptom relief has also notably improved as newer derivatives have been developed, increasing from between 2 and 6 h for naphazoline to 8–12 h for oxymetazoline (Table 3) (Passali et al., 2006). Interestingly, the shorter acting derivatives, including naphazoline and tetrahydrozoline have been observed to cause reactive hyperaemia as soon as 8 h after application (Hochban et al., 1999). The presence of reactive hyperaemia has the potential to lead to patients misusing a nasal decongestant, in an attempt to provide further relief from the ongoing sensation of nasal congestion (Russo et al., 2023). Consequently, this increased duration of action means patients require less frequent dosing, thereby reducing the risk of experiencing unwanted side effects (Wang et al., 2024; Passali et al., 2006).

The improved symptom relief observed with new-generation nasal decongestants is a reflection of changes to interactions with the adrenergic receptors. Although xylometazoline was more effective than previously designed nasal vasoconstrictors, the modification to create oxymetazoline further improved potency at some adrenergic receptors. Whilst xylometazoline acts as full agonist at the α_2_B-adrenergic receptors, oxymetazoline is not only a full agonist but also has a seven-fold higher potency, in addition it is a weak, partial agonist at α_1_A-adrenergic. Furthermore, oxymetazoline has a higher affinity for the α_1_A-adrenergic receptors compared to xylometazoline, which has a higher affinity to α_2_B-adrenergic receptors. These differences have the potential to help explain the faster onset of action and different dosage requirements compared to other early generation imidazolines (Haenisch et al., 2010; Horie et al., 1995; Jones, 2021).

### 3.4 Safety considerations

Imidazoline nasal decongestants have an improved efficacy and safety profile compared to other nasal decongestants such as phenylephrine, often making them the decongestant of choice (Cartabuke et al., 2021). This safety profile is supported through the limited published evidence of acute intoxication and specifically fatalities. As with any medication, there is a risk of developing acute side effects, which in the case of imidazoline nasal decongestants are typically minor and short lived. However, a chronic side effect in the form of rhinitis medicamentosa (RM) can develop if taken for a prolonged period or if the recommended daily dose is exceeded (Passali et al., 2006). During normal use, commonly observed side effects include sneezing, a runny nose and burning or stinging sensations, while less common side effects can include wheezing, dizziness, headaches and palpitations (Table 4) (Patel et al., 2021). These acute and chronic risks will be discussed in further detail below, whilst RM will be explored in greater depth in the second part of our literature review series and below in 3.4.2.

**TABLE 4:** Examples of some of the potential side effects that can be experienced with the most widely available nasal decongestant sprays.

Common side effects
Sneezing Burning/Stinging sensation Nasal dryness/Irritation Mild headaches

Less common side effects
Dizziness Severe headaches Increased blood pressure* Irregular/Rapid heartbeat* Insomnia/Anxiety* Rhinitis medicamentosa Drowsiness* Breathing difficulties*

*Represents side effects that typically occur due to systemic absorption, ingestion/overdose or sensitivity and are less likely to occur when taken as prescribed (Wang et al., 2024; Jones, 2021; Gussow, 2020; Lake, 1946; Pasini and Massara, 1958; Corboz et al., 2008; Greene, 2005; Ahsanuddin et al., 2021).

#### 3.4.1 General toxicology

The first cases of acute toxicity after excessive self-medication with imidazoline nasal sprays were reported in 1946. Regardless of the imidazoline derivative used, the observed effects of intoxication are similar, and can include central nervous system and respiratory depression, alongside reduced heart rate and fluctuations in blood pressure. However, the dose required for intoxication to occur is often not well defined and varies between imidazoline derivatives. The onset of symptoms of intoxication are rapid and typically resolve within 24 h with suitable medical care (Table 5) (Cartabuke et al., 2021; Uehara et al., 2024; Oishi et al., 2024; Vitezić et al., 1994; van Velzen et al., 2007; Spiller and Griffith, 2008; Al-Abri et al., 2014; Musshoff et al., 2014; van Stralen et al., 2023; Nordt et al., 2016; Gussow, 2020).

**TABLE 5:** Overview of the effects caused by intoxication with the most commonly used imidazoline nasal decongestants. The intoxication dose reflects the variability of reporting in the literature, whilst route, effect, onset and resolution of the intoxication are similar for all imidazoline nasal decongestants. The effects listed here are not exhaustive, other effects may also be observed (Cartabuke et al., 2021; Uehara et al., 2024; Oishi et al., 2024; Vitezić et al., 1994; van Velzen et al., 2007; Spiller and Griffith, 2008; Al-Abri et al., 2014; Musshoff et al., 2014; van Stralen et al., 2023; Nordt et al., 2016).

Imidazoline derivative	Naphazoline	Tetrahydrozoline	Xylometazoline	Oxymetazoline
Intoxication dose	0.05–0.3 mg/kg	67–102 mg/kg 2–3 mL in a child	No effect observed at <0.4 mg/kg	1–2 mL in a child of 0.05% solution
Route of dose	Ingestion (0.1 mg/kg in babies, 0.3 mg/kg children over 2) Intranasal (0.05 mg/kg)	Ingestion Intranasal	Ingestion Intranasal	Ingestion Intranasal (in children)
Effect	CNS depression – including effects such as miosis, convulsions, drowsiness, loss of consciousness and potential coma Cardiovascular involvement – including effects such as bradycardia, tachycardia and changes to BP (initial hypertension followed by hypotension) Respiratory depression Other effects include vomiting, hypothermia, loss of muscle tone and paleness			
Onset of symptoms of intoxication	Within 2 h	Within 2 h	Within 2 h	Within minutes
Resolution of symptoms of intoxication	12–36 h	24–36 h	20 h	24 h

These initial instances of intoxication with naphazoline reflected the relatively low efficacy of the drug and the need to take frequent doses to maintain the decongestive effect (Mertins, 1947). This frequent usage increases the risk of the observed acute toxicity, especially if delivered incorrectly resulting in accidental ingestion or if given to children where a more pronounced effect is seen compared to adults (Wenzel et al., 2004). Similar observations can also be made with tetrahydrozoline (Brainerd and Olmsted, 1956; Chusid et al., 1956). However, being widely available as an OTC drug, tetrahydrozoline use has been implicated in more illicit practices. Albeit infrequently, tetrahydrozoline has been identified as a drug which can be used to facilitate sexual assault, linked to creating false negatives in urine drug tests and in attempted suicides and murders (Menshawey and Menshawey, 2024; Sosa, 2024)

With xylometazoline it took nearly 30 years before any mention of acute toxicity appeared in the literature (Vanezis and Toseland, 1980). Examples of intoxication are limited, with those that exist, typically due to accidental oral consumption or as a consequence of dosage errors (Musshoff et al., 2014). Xylometazoline as with tetrahydrozoline has been used for illicit means, as inhalation can cause rewarding psychoactive effects (Anand et al., 2008). Oxymetazoline has a safety record similar to xylometazoline, with little evidence of acute toxicity documented in the literature when taken correctly. However, as with other nasal decongestants the risk of toxicity is higher in children and adolescents compared to adults, especially when systemic absorption is considered (Nordt et al., 2016). Incidentally, oxymetazoline has not been linked to use for illicit means. Furthermore, the high efficacy, longer duration of action and increased affinity and potency towards the α-adrenergic receptors, has resulted in it becoming the most widely used nasal decongestants across the United States (Trinh et al., 2024).

#### 3.4.2 Implications of imidazoline misuse–rhinitis medicamentosa and addiction

General side effects and acute risks of intoxication are not the only problems which can occur with the use of imidazoline nasal decongestants, specific chronic risks can also occur when taken incorrectly. Within 3 years of naphazoline becoming available for the treatment of nasal congestion, a very specific side effect started to be reported. This took the form of nasal congestion without rhinorrhoea, sneezing or post-nasal drip which continued to occur after initial successful treatment with a nasal decongestant. Later termed rhinitis medicamentosa (RM), this specific side effect is not unique to naphazoline and is seen in many nasal decongestants (Kuzminov et al., 2018; Ramey et al., 2006). In 1945 two different observations were made about the misuse of nasal decongestants of differing classes. Prolonged use of naphazoline was linked to the maintenance or aggravation of nasal congestion, whilst excessive use of sympathomimetic amine based nasal decongestants, e.g., ephedrine was linked to secondary vasodilatation (Kully, 1945; Feinberg, 1945). These observations were the foundation for the development in recognising the side effect now known as RM or rebound congestion. Initially it was not directly linked to imidazoline based nasal spray misuse however, this changed when tetrahydrozoline was mentioned in direct relation to RM (Lake, 1946; Pasini and Massara, 1958). RM has since become synonymous with abuse or excessive use of nasal decongestants but can also be attributed to prolonged duration of use. This misuse of a nasal decongestant results in rebound swelling of the nasal mucosa that gives a sensation of continued stuffiness within the nose even after treatment (Chodirker, 1981; Graf and Juto, 1995). Interestingly, RM has since been shown to occur to differing extents depending on the imidazoline derivative used and the length of time it is continuously used for (Black and Remsen, 1980; Wang et al., 2024). As a consequence of the continued nasal stuffiness experience in RM, patients continue to use nasal decongestants beyond the recommended treatment period.

This frequent need to readminister the nasal spray to provide the desired decongestion effect, resulted in the concept of ‘privinism’, or addiction to nasal decongestants being proposed. This concept stems from the idea that patients continue to take the nasal decongestant beyond the recommended maximum time limit and at increased dosages or frequency in an attempt to relieve the symptoms of nasal congestion. As usage continues, the relief provided by each dose becomes less pronounced resulting in the patient further increasing frequency or dosage in order to provide a similar level of symptom relief, thereby creating a cyclical effect and thus dependency on the nasal spray (Mosges et al., 2017; Graf et al., 1999; Maunsell, 1959). Interestingly, prolonged use of imidazolines reduces their effectiveness, treatment of nasal congestion with xylometazoline for 30 days has been shown to reduce the effectiveness of each application from more than 9 h to approximately 5 h. This decrease in effectiveness has been linked to either a downregulation in the number of α-adrenergic receptors or reduced affinity of the drug to the receptors rather than the development of a tolerance to the drug however, these mechanisms have not yet been fully elucidated (Graf and Juto, 1995). These ideas will be discussed in the second part of our review series which focuses specifically on different aspects of RM.

#### 3.4.3 Imidazoline nasal decongestant use in children

When used as directed, imidazoline nasal decongestants are considered safe and typically only cause minor side effects. However, as with any drug there is the potential for errors in delivery, misuse or contraindicators which increase the risk of developing serious adverse effects. One major risk factor for developing serious adverse effects is age, specifically the use of imidazoline nasal decongestants in paediatric care. Consequently, many formulations of imidazoline nasal decongestants are not recommended for use in children under 6, although this varies depending on country and formulations (Cartabuke et al., 2021; van Stralen et al., 2023; Nordt et al., 2016; Scadding, 2008; European Medicines Agency, 2021). Where treatment is available for children younger than 6, formulation concentrations of oxymetazoline and xylometazoline are reduced to half the adult dose, 0.025% and 0.5% respectively, to treat children aged 2–6 years and then halved again, 0.01% and 0.025% respectively to treat children under 2. In addition, these products are not as readily available as adult formulations and often require medical advice to be sought before use (Cartabuke et al., 2021; van Stralen et al., 2023; European Medicines Agency, 2021; European Medicines Agency, 2024). However, there is limited evidence to suggest that the use of nasal decongestants in children is beneficial to the reduction of symptoms related to acute sinusitis (Runkle, 2016). Nevertheless, although 0.05% oxymetazoline is not recommended for use in the under 6 population, it is on occasion used off-label by medical professionals in the short term, if the benefits outweigh the risk (Cartabuke et al., 2021). Similarly, given their ease of access without a prescription, parents may use 0.05% formulations in young children without medical guidance and against recommendations (Fabi et al., 2009). However, lack of pharmacokinetic studies regarding paediatric use means extreme caution needs to be exercised when given in children to reduce the risk of serious adverse side effects (Cartabuke et al., 2021) and where possible the lowest effective concentration formulations should be utilised.

The risk of severe adverse effects in children is due to an increased likelihood of systemic absorption when compared to adults, due to accidental ingestion or swallowing “run-off” from the posterior oropharynx (Nordt et al., 2016). An issue that is further compounded by the lack of consensus around suitable paediatric doses and treatment duration, coupled with ease of access without medical guidance (Bucaretchi et al., 2003). The risk of systemic absorption often relates to position of the patient and the type of device used to deliver the treatment, which can directly impact the dosage delivered to the patient. For example, the use of nasal decongestants in inverted spray bottles in patients in a supine position can result in the release of 0.5–1.5 mL per spray, compared to 0.03 mL being released per spray when used in an upright position (Cartabuke et al., 2021). Given that only 0.15 mL of solution can be absorbed nasally, the resulting excess can drain into the oropharynx and be ingested (Nordt et al., 2016). As such the use of nasal decongestants in a supine position requires careful management to avoid such consequences. A potentially safer alternative for administering nasal decongestants in children during surgery, suggested in one study is to soak pledgets in a known volume of the treatment. This reduces the risk of accidental ingestion and allows for slower absorption across the nasal mucosa potentially due to the vasoconstrictive effect (Cartabuke et al., 2019). However, this alternative application relates directly to the use of nasal decongestants in a surgical or medically managed setting and does not solve the issue regarding ease of access or the decision by some individuals to use them in an unrecommended manner. As such, alternative measures have been introduced to reduce the acute risk from application in the supine position and accidental ingestion, particularly by paediatric patients under the age of 6, but could be applicable to any age group, these will be discussed in more detail in 3.4.5

#### 3.4.4 Contraindicators for imidazoline nasal decongestant use

Whilst the use of imidazoline nasal decongestants is not necessarily problematic in the elderly, the likelihood of having comorbidities which can increase the risk of developing serious adverse effects may be higher, therefore caution should be exercised when using them in the elderly (Slavin, 2009). Thus, it is worth noting that the use of nasal decongestants is a contraindicator in individuals with existing hypertension. The vasoconstrictive action of imidazoline based nasal decongestants has the potential to increase blood pressure further, due to the non-selective targeting of the α-adrenoreceptors beyond the nasal mucosa, in these individuals it can also confer a slight increase in the risk of stroke (Wang et al., 2024; Corboz et al., 2008). However, it is worth noting that this increased risk of stroke is typically linked to not only an existing cardiovascular risk but also chronic use, whilst a similar association is less clear when the correct dosage is utilised (Patel et al., 2021; Leupold and Wartenberg, 2011; Grimaldi-Bensouda et al., 2021; Lafaurie et al., 2020). Furthermore, accidental ingestion or excess use of imidazolines has cardiac consequences beyond hypertension, and whilst symptoms such as tachycardia are rare with these treatments, some individuals instead experience bradycardia due to increased vagal tone (Wang et al., 2024; Gussow, 2020).

#### 3.4.5 Safety measures to reduce acute risk

To mitigate against some of the acute risk associated with incorrectly used imidazoline nasal decongestants, some manufacturers have modified delivery systems to help minimise the risk of accidental ingestions. One change includes the use of metered dose sprays or metered drops in place of nasal drops or squeeze bottles. The disadvantage of using nasal drops or squeeze bottles is the high likelihood of inaccurate doses and limited control over delivery, which increases the risk of overdose via accidental ingestion, systemic absorption and adverse side effects. Conversely, metered sprays have the advantage of providing a reproducible dose volume and delivery, which prevents underdosing, thereby ensuring a therapeutic dose is delivered each time the nasal decongestant is used (Bharagava et al., 2025). Additionally, due to the manner in which metered sprays are deposited in the nasal cavity they are less likely to flow than nasal drops, thus being harder to swallow (Wang et al., 2024). Consequently, metered dose sprays have become the dominant delivery system for nasal drugs (Thorat, 2016). Furthermore, although not directly related to imidazoline nasal decongestants, it has been suggested that nasal sprays are preferable for the delivery of intranasal drugs in paediatric care compared to nasal drops. This reflects a higher level of cooperation and overall better patient condition when nasal sprays are used, with no impact on the side effects experienced. Moreover, although the use of both application methods can cause discomfort, nasal sprays are as well tolerated by the patient as nasal drops and therefore provide a better method of application than drops (Ljung and Andréasson, 1996). However, the use of sprays does rely on a certain level of cooperation from the patient, which is not always easy when younger children are involved. Thus, for low dose paediatric formulations for use in the under 2s, an alternative method of instilling the nasal decongestant is available, in the format of metered droppers. Metered droppers are designed to instill a single drop of nasal decongestant into the nostril opening of a young child, whilst in the supine position. This not only reduces the risk of accidentally administering a larger dose than recommended, lowering the likelihood of unwanted ingestion, but also removes the potential for damaging the mucosal membrane of the nasal passage as the device does not need to be placed inside the nostril to enable correct delivery. Furthermore, these devices use the lowest effective dose of oxymetazoline, which is already lower than for other imidazoline nasal decongestants thus further reducing the risk of adverse side effects (European Medicines Agency, 2021; European Medicines Agency, 2024; Bergner and Tschaikin, 2005; National Drug Formulary. Minis try of Health Singapore, 2021). In one small prospective study, these metered drops showed a high level of efficacy tolerability in paediatric patients, with no adverse effects reported. Additionally, they showed an improvement in the rhinitis symptoms including nasal breathing, which consequently helped reduce difficulties in feeding (Bergner and Tschaikin, 2005). However, with the limited studies around the pharmacokinetics of nasal decongestant usage in paediatrics, it is difficult to understand if systemic absorption may occur and the risk this poses to the patient.

Whilst metered dose sprays and metered drops reduce the risk of delivering an excessively large dose, especially in the supine position, they are not designed to be inherently child resistant, as there is the possibility that a child could ingest the imidazoline by drinking from a bottle. Therefore, although an overdose via correct usage is unlikely, it would still be possible for a child to accidentally ingest large quantities of the imidazoline. Consequently, in a move to address this risk, the US government under the Poison Prevention Packaging Act 1970 added any product containing more than 0.08 mg of imidazoline to a list of substances that must have child-resistant packaging (Ravindra et al., 2024). This child-resistant packaging is not designed to be unopenable by young children but rather slows their ability to open the packaging, whilst ensuring that it remains elderly friendly, thus reducing the likelihood of accidental ingestion of a potentially dangerous substance (Ravindra et al., 2024; Bakshi and Patel, 2025). However, this move has currently not been replicated globally, and there is still a risk that accidental ingestion of nasal sprays could occur. Arguably it would be worth considering the expansion of this safety feature to help further reduce the potential for accidental ingestion.

Although the development of newer imidazoline nasal decongestants, such as xylometazoline and oxymetazoline, has provided a notable safe, less open to abuse and more effective treatment than previous nasal vasoconstrictors, there are numerous aspects of these treatments which would benefit from further study. Owing to limited number of pharmacokinetic studies, particularly in children, the uptake of the treatment at the nasal mucosa and its downstream action is not fully understood (Cartabuke et al., 2021). Similarly, the mechanism involved in rebound congestion and the development of RM require further elucidation (Graf and Juto, 1995). Thus, the potential acute risk associated with nasal decongestant use in children or through accidental ingestion, and the chronic risk associated with prolonged use, all continue to be problematic and as such would benefit from further research to understand the mechanisms involved, to determine whether such issues can be resolved. Whilst this risk should not be underestimated, the widespread use of nasal decongestants, coupled with the length of time they have been available on the market, does not appear to translate to large numbers of incidences of severe adverse effects and specifically fatalities being described in the literature. These observations of limited numbers of adverse effects support the argument that imidazoline nasal decongestants are safe when used as recommended. Indeed, a recent systematic review concluded that when used as directed, oxymetazoline does not lead to rebound congestion (Malik et al., 2025). However, the importance of pharmacovigilance should not be underestimated as a means to monitor instances of adverse effect and continue to support the safety profile of these nasal decongestants, the role of pharmacovigilance will be discussed in 4.3. Furthermore, whilst the aforementioned issues relating to risk cannot immediately be solved, it is important to provide clear, concise and easy to remember information to patients on the correct use of nasal decongestants, as an optimally delivered dose can act more effectively and may help reduce the potential for unwanted side effects.

## 4 The importance of correct usage of nasal decongestants

### 4.1 Patient guidance

**FIGURE 6:**
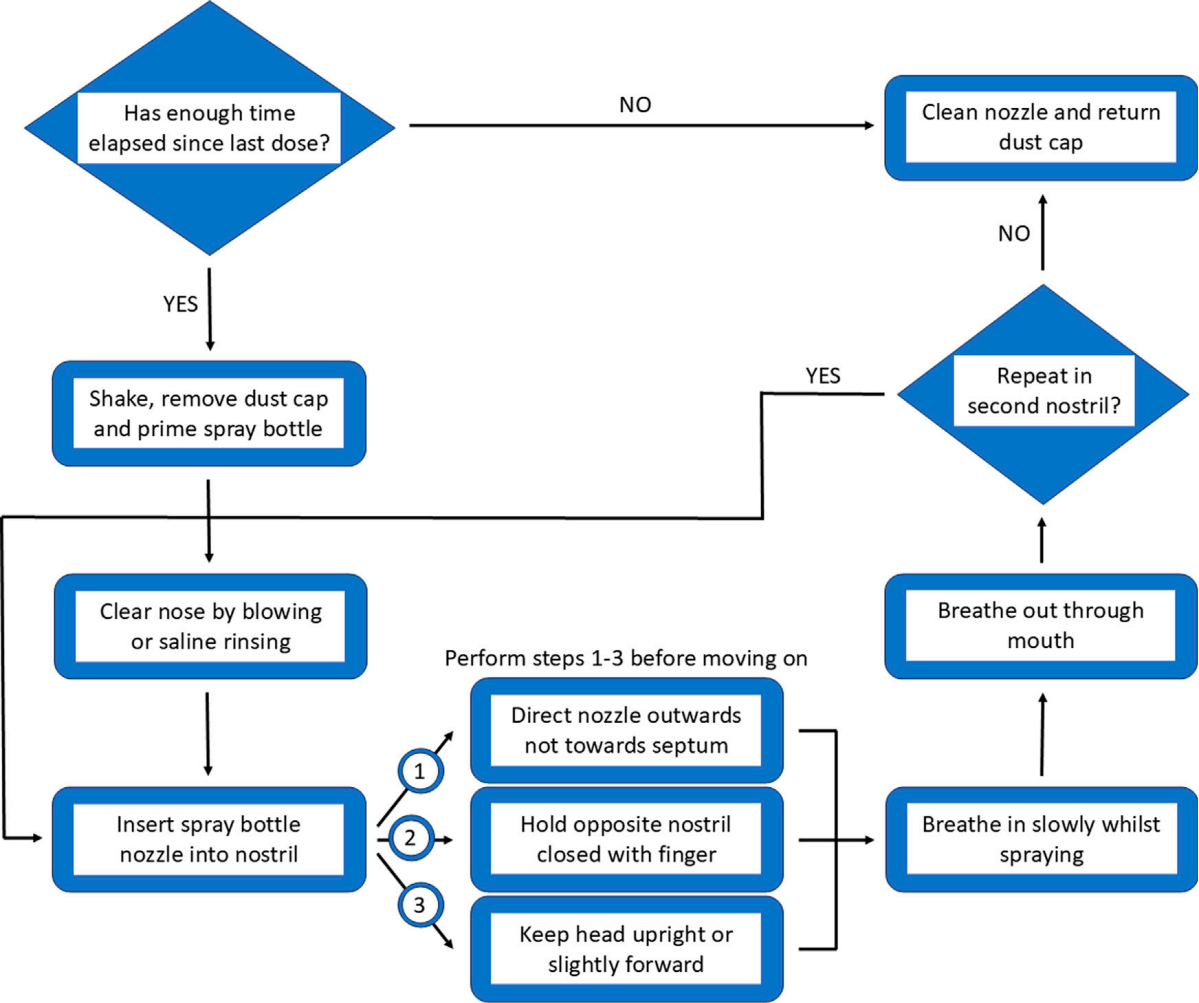
Flow chart depicting the steps for optimal use of a nasal decongestant.

A general internet search for “how to use a nasal spray correctly?” provides a wealth of videos and images from a variety of sources, which aim to portray the best method to achieve the most effective results. One method of providing information to patients is through the inclusion of leaflets alongside medication however, this is not mandated worldwide thus there is the possibility that patients may not be made aware of potential risks (Young et al., 2017). Moreover, where leaflets are received, they often differ in terms of readability (Okoro, 2022). Whilst receiving an information leaflet alongside medication appears to be beneficial, with most patients taking the time to read them the first time they use the medication, they are less frequently checked when subsequent packs are opened thus updated information can be missed. Additionally, the language used within these leaflets can be difficult to access for some patients due to factors such as overwhelming amounts of information, technical language usage and relevance to the patient (Barrias et al., 2024). The use of pictorial representations and visual aids may facilitate patient understanding and comprehension rather than text format alone. Whilst, this has little impact on adherence, it has the potential to positively improve health outcomes (Schubbe et al., 2020). Given the potential risk which may arise from the accidental ingestion of imidazoline nasal decongestants, it would be worth considering simplifying the information received by patients. Making use of a combination of visual aids and text could help facilitate correct use, improving not only efficacy but also tolerability. An overview of how to approach the optimal use and delivery is described in Figure 6.

### 4.2 Optimal delivery

Perhaps the most important element of the correct use of nasal decongestant sprays is delivery to the correct area on the nasal passages. The anatomy of the nose means the nasal passages are an ideal site for the application of topical therapeutics, as the extensive vasculature and large surface area provides optimal opportunity for absorption. However, the narrowness of nostril and presence of the nasal valve creates a flow limiting region. Outside of the nose, nasal sprays often produce a cone shaped plume however, inside the nose this effect is impeded by the nasal anatomy, meaning that nasal sprays cannot disperse in the same manner inside the nose as it would if sprayed unimpeded outside nose. Interestingly, even the design of the applicator can impact the effectiveness of the delivery system. The complexity of the nasal anatomy means the correct delivery within the nostril is imperative if maximum benefit is going to be achieved (Tong et al., 2016; Djupesland and Mahmoud, 2014). To optimise delivery, the nasal spray applicator should be placed around 10 mm into the nostril and aimed centrally with the spray angled slightly towards the outermost surface of the nasal mucosa as opposed to the septal area. This not only helps the decongestant penetrate deeper into the nasal cavity as it is not impeded by the nasal anatomy, it also helps to increase the delivery of the active ingredient to the ciliated epithelial cells which can more rapidly absorb the treatment, compared to the non-ciliated epithelial cells around the anterior septal region. Furthermore, using this angle of spray also reduces the likelihood of excess irritation. The head should be positioned neutrally or tilted slightly forward to reduce the chances of accidental ingestion, with calm breathing to maximise distribution within the nasal cavity. Whilst this advice is provided to patients via information leaflets, the details vary. Consequently, this inconsistent detail when coupled with incorrect application leads to suboptimal drug delivery, reducing effectiveness and increasing the risk of side effects (Tong et al., 2016; de Boer et al., 2020; Rollema et al., 2019). Whilst overall, correct delivery increases the efficacy of nasal decongestants, this comes with a caveat that individual differences in nasal anatomy and variations in nasal spray design may impact what is considered optimal delivery, thus some minor adjustments may be required by the patient to provide optimum relief from nasal congestion (Tong et al., 2016). Given the inconsistency in the information provided with nasal sprays it is not surprising that patients are at risk of experiencing side effects from their suboptimal use.

### 4.3 Pharmacovigilance

Although the likelihood of developing side effects is reduced through the correct application and duration of use of imidazoline nasal sprays, as with all drugs they are subject to monitoring for adverse effects through the various pharmacovigilance databases which exist worldwide. These databases which have been operational since the 1960s include reported adverse drug reactions (ADR) by healthcare providers or the patient themselves (Bate et al., 2018; Wa et al., 2019). Pharmacovigilance is conducted on a global scale by the World Health Organisation (WHO) via VigiBase. Additionally, most high income countries also operate their own databases, with the European Union’s Eudravigilance and the USA’s FDA Adverse Event Reporting System (FAERS) being recognised as world leaders (Khan et al., 2023). These databases are particularly important for monitoring not only ADRs experienced by adults, but also paediatric ADRs due to the off-label use of some drugs. Monitoring via these databases enables patterns in ADRs to be identified promptly, activating further investigations and where necessary results in changes to prescribing recommendations or withdrawal of a drug (Arnott et al., 2013). When the four main imidazoline nasal decongestants are searched in WHO-VigiBase via the public-facing WHO-VigiAccess, around 15,000 ADRs from a total of over 40 million records are presented, these are classified into 27 categories and represent ADRs reported from over 180 member countries and territories. Overall, these ADRs are predominately observed relatively equally in males and females, aged 18–64 years or in the unknown age group, and the highest number of events are documented in either Europe or America (World Health Organisation, 2025; Uppsala Monitoring Centre, 2025). Whilst there has been an increase in the number of recorded events since around 2010, this potentially reflects not only how widely available these treatments are but also changes to how ADRs are recorded. Factors such as improved awareness of reporting and patients being able to self-report, is likely to have increased these numbers rather than being due to changes in the safety profile of the nasal decongestant (Trinh et al., 2024; Wang et al., 2024; Margraff and Bertram, 2014; Sienkiewicz et al., 2021; Valinciute-Jankauskiene and Kubiliene, 2021; Zatovkaňuková and Slíva, 2024). However, the number of ADRs which are reported are likely only a small proportion of those experienced, due to either lack of awareness of reporting schemes or time (Eland et al., 1999). Nevertheless, it is worth noting that although ADRs can be experienced, including those which have the potential to be life-threatening, there is limited literature or real-world evidence through pharmacovigilance of very serious or fatal outcomes relating to imidazoline nasal decongestant usage, particularly when used correctly (Lafaurie et al., 2020). Furthermore, it is worth noting that there is also no differentiation on the route of administration for the imidazoline derivative included in the above search, as it is performed based on the active ingredient. As such, it could not only include intranasal applications, but also accidental ingestion or ocular applications. Nevertheless, given the ease of access and widespread use of imidazoline nasal decongestants, these observations in terms of limited serious ADRs further support the safety of these treatments as a method to provide relief from nasal congestion.

## 5 Recent developments and future insights

### 5.1 Computational fluid dynamics

Understanding how the nasal decongestant is deposited, distributed and thus where and how quickly it works within the nasal passages may be a useful way of improving efficacy of the imidazolines already in use. Furthermore, this could be used to help further improve the delivery technology and education of patients on how best to administer the dose. MRI and computed tomography scans have been used to create 3D images of the nasal passages and airflow is simulated using computational fluid dynamics. Importantly, the effectiveness of both xylometazoline and oxymetazoline in improving nasal congestion have been shown in studies utilising computational fluid dynamics (Kishore et al., 2015; Xiao et al., 2021; Hamdan et al., 2024).

Further computational fluid dynamic models have been used to investigate the deposition of drugs delivered via nasal sprays within the nasal cavity. Using these models the location of decongestant deposition within the nasal cavity has been determined. Furthermore, the importance of particle size and spray angles have been investigated in healthy individuals with normal but varying nasal vestibule morphology or patients undergoing nasal surgery (Sicard and Frank-Ito, 2024; Liu et al., 2025). Utilising these models could be used to further improve the efficacy and safety of nasal decongestants.

### 5.2 Structure activity and *in silico* studies

Although the imidazolines currently used in nasal decongestants are fast acting and effective when used correctly, they could be developed further by improving these properties, investigating binding and reducing tachyphylaxis associated with RM (Vaidyanathan et al., 2010). Past publications have explored the structure activity relationships of the adrenergic receptors including the α_1_ subtype (Perez, 2007; Wu et al., 2021). For example, with the imidazoline cirazoline, oxygen in the side chain is essential for activity at the α_1_ adrenergic receptor whilst the cyclopropyl ring plays a role in selectivity (Pigini et al., 2000). Some attention has shifted to investigating downstream biased signalling initiated by different adrenergic receptor agonists including the imidazolines (Proudman and Baker, 2021) which may be an interesting and important area for understanding agonist activity and possible involvement with RM. However, there is a lack of recent studies in this area or using *in silico* approaches to investigate and design further new generation imidazolines for nasal decongestion. Investigating structure activity relationships or applying recent advances in our knowledge of signalling pathways to imidazolines research may be an important way of developing understanding of imidazoline pharmacodynamics leading to further improvements in efficacy and safety.

## 6 Conclusion

Although first identified nearly 150 years, the last 75 years has seen an extensive and complex development of the imidazolines, driven by a need to create derivates with higher efficacy and fewer side effects. This continued drive for improvement has given rise to a new generation of imidazolines that demonstrate enhanced effectiveness, requiring fewer doses for the same therapeutic effect. These newer molecules typically have fewer side effects, improved tolerability, a subjective faster onset of action, longer duration and greater α-adrenergic receptor affinity and potency (Schafer et al., 1992; Druce et al., 2018; Reinecke and Tschaikin, 2005). Why imidazolines were first trialled as a treatment for nasal congestion remains elusive in the literature, nevertheless these treatments have proven to be a mainstay in decongestive medications for over 75 years, continuing to be sought after by individuals managing seasonal and chronic nasal congestion. The advent of clonidine transformed hypertension treatment and facilitated a deeper understanding of imidazoline mechanisms of action and related receptors. However, there still exists a number of gaps relating to the mechanisms, especially when their use as nasal decongestants are considered. The pharmacokinetics of these nasal decongestants would benefit from further elucidation, particularly in relation to their use in paediatric patients. Furthermore, although newer imidazoline-derived nasal decongestants have been shown to be safe when used as recommended, issues with tolerability and side effects continue to persist for some individuals, particularly, when linked to overuse or misuse. Thus, there is a need to continue to review not only pharmacovigilance data for evidence of these ADRs but also consider how and why these issues persist, and what can be done to further reduce their burden. As such, patient education should be paramount as a mechanism to highlight not only the importance of correct usage, but also the benefit of using the lowest effective dose for the shortest period of time to reduce the risk of both acute and chronic side effects. Where chronic risks are concerned, RM is a significant burden faced by patients using imidazoline nasal decongestants, owing to the widespread use of imidazolines. This issue has only been discussed briefly within this review and it would be beneficial to explore the underlying mechanism of this side effect further. Therefore, we will discuss the mechanisms behind this phenomenon and the differences amongst commonly used imidazoline derivates in our subsequent literature review.

## References

[B1] Abelson M. B. Butrus S. I. Weston J. H. Rosner B. (1984). Tolerance and absence of rebound vasodilation following topical ocular decongestant usage. Ophthalmology 91 (11), 1364–1367. 10.1016/s0161-6420(84)34140-9 6514304

[B2] Ahsanuddin S. Povolotskiy R. Tayyab R. Nasser W. Barinsky G. L. Grube J. G. (2021). Adverse events associated with intranasal sprays: an analysis of the food and Drug Administration database and literature review. Ann. Otol. Rhinol. Laryngol. 130 (11), 1292–1301. 10.1177/00034894211007222 33813873

[B3] Åkerland A. Klint T. Olén L. Rundcrantz H. (1989). Nasal decongestant effect of oxymetazoline in the common cold: an objective dose-response study in 106 patients. J. Laryngology and Otology 103 (8), 743–746. 10.1017/S0022215100109958 2671220

[B4] Al-Abri S. A. Yang H. S. Olson K. R. (2014). Unintentional pediatric ophthalmic tetrahydrozoline ingestion: case files of the medical toxicology fellowship at the University of California, San Francisco. J. Med. Toxicol. 10 (4), 388–391. 10.1007/s13181-014-0400-9 24760708 PMC4252297

[B5] Anand J. S. Salamon M. Habrat B. Scinska A. Bienkowski P. (2008). Misuse of xylometazoline nasal drops by inhalation. Subst. Use Misuse 43 (14), 2163–2168. 10.1080/10826080802344625 19085441

[B6] Arnott J. Hesselgreaves H. Nunn A. J. Peak M. Pirmohamed M. Smyth R. L. (2013). What can we learn from parents about enhancing participation in pharmacovigilance? Br. J. Clin. Pharmacol. 75 (4), 1109–1117. 10.1111/j.1365-2125.2012.04441.x 22905902 PMC3612729

[B7] Bakshi A. Patel P. (2025). “Poison prevention packaging act,” in StatPearls (Treasure Island (FL): StatPearls Publishing). Available online at: https://www.ncbi.nlm.nih.gov/books/NBK572141/. 34283507

[B8] Barrias A. Di Lauro E. Dunnett S. Flick F. Smerdka P. Wardle F. (2024). European pharmaceutical industry medical information: a role to play in the provision of medicine-related information to patients. Pharm. Med. 38 (6), 399–405. 10.1007/s40290-024-00534-x 39535583 PMC11625064

[B9] Bate A. Reynolds R. F. Caubel P. (2018). The hope, hype and reality of big Data for pharmacovigilance. Ther. Adv. Drug Saf. 9 (1), 5–11. 10.1177/2042098617736422 29318002 PMC5753994

[B10] Bende M. Pipkorn U. (1987). Topical levocabastine, a selective H1 antagonist, in seasonal allergic rhinoconjunctivitis. Allergy 42 (7), 512–515. 10.1111/j.1398-9995.1987.tb00374.x 2891316

[B11] Bergner A. Tschaikin M. (2005). Schnupfenbehandlung bei Babys und Kleinkindern. Dtsch. Apoth. Ztg. -stuttgart-. 145 (44), 97–98.

[B12] Bharagava S. Medikeri G. Narendran R. Ravichandran S. K. Agarwal V. Ali A. (2025). Selecting the correct dosage form for topical nasal decongestants: an expert opinion. J. Assoc. Physicians India 73 (5), e11–e15. 10.59556/japi.73.0962 40553527

[B13] Bhatnagar A. Sharma P. Kumar N. (2011). A review on “Imidazoles”: their chemistry and pharmacological potentials. Int. J. PharmTech Res. 3 (1), 268–282.

[B14] Black M. J. Remsen K. A. (1980). Rhinitis medicamentosa. Can. Med. Assoc. J. 122 (8), 881–884. 6154514 PMC1801634

[B15] Boora K. (2007). “Antazoline,” in xPharm: the comprehensive pharmacology reference. Editors Enna S. J. Bylund D. B. (New York: Elsevier), 1–4.

[B16] Bousquet P. (1995). Imidazoline receptors: from basic concepts to recent developments. J. Cardiovasc Pharmacol. 26 (Suppl. 2), S1–S6. 10.1097/00005344-199506262-00001 8642798

[B17] Bousquet P. Hudson A. Garcia-Sevilla J. A. Li J. X. (2020). Imidazoline receptor system: the past, the present, and the future. Pharmacol. Rev. 72 (1), 50–79. 10.1124/pr.118.016311 31819014

[B18] Brainerd W. K. Olmsted R. W. (1956). Toxicity due to the use of tyzine hydrochloride. J. Pediatr. 48 (2), 157–164. 10.1016/s0022-3476(56)80161-3 13295960

[B19] Bucaretchi F. Dragosavac S. Vieira R. J. (2003). Acute exposure to imidazoline derivatives in children. J. Pediatr. Rio J. 79 (6), 519–524. 10.2223/jped.1112 14685449

[B20] Bylund D. B. (1985). Heterogeneity of alpha-2 adrenergic receptors. Pharmacol. Biochem. Behav. 22 (5), 835–843. 10.1016/0091-3057(85)90536-2 2989948

[B21] Cartabuke R. S. Anderson B. J. Elmaraghy C. Rice J. Tumin D. Tobias J. D. (2019). Hemodynamic and pharmacokinetic analysis of oxymetazoline use during nasal surgery in children. Laryngoscope 129 (12), 2775–2781. 10.1002/lary.27760 30786035 PMC6702099

[B22] Cartabuke R. Tobias J. D. Jatana K. R. Section On A. Pain M. S. O. O.-H. Neck S. (2021). Topical nasal decongestant oxymetazoline: safety considerations for perioperative pediatric use. Pediatrics 148 (5), e2021054271. 10.1542/peds.2021-054271 34607935

[B23] Chatelet J. (1965). Clinical trials of oxymetrazoline hydrochloride. Prog. Med. Paris. 93 (8), 321–323. 5833240

[B24] Chodirker W. B. (1981). Rhinitis medicamentosa. Can. Med. Assoc. J. 124 (4), 370–372. 6163517 PMC1705259

[B25] Chusid E. Lehr D. Levy W. (1956). Severe respiratory depression following nasal instillation of tetrahydrozoline hydrochloride. J. Pediatr. 48 (1), 66–69. 10.1016/s0022-3476(56)80119-4 13295949

[B26] Connell J. T. Linzmayer M. I. (1987). Comparison of nasal airway patency changes after treatment with oxymetazoline and pseudoephedrine. Am. J. Rhinology 1 (2), 87–94. 10.2500/105065887781693358

[B27] Corboz M. R. Rivelli M. A. Mingo G. G. McLeod R. L. Varty L. Jia Y. (2008). Mechanism of decongestant activity of alpha 2-adrenoceptor agonists. Pulm. Pharmacol. Ther. 21 (3), 449–454. 10.1016/j.pupt.2007.06.007 17869148

[B28] Costa V. M. Grando L. G. R. Milandri E. Nardi J. Teixeira P. Mladenka P. (2022). Natural sympathomimetic drugs: from pharmacology to toxicology. Biomolecules 12 (12), 1793. 10.3390/biom12121793 36551221 PMC9775352

[B29] Dardonville C. Rozas I. (2004). Imidazoline binding sites and their ligands: an overview of the different chemical structures. Med. Res. Rev. 24 (5), 639–661. 10.1002/med.20007 15224384

[B30] de Boer M. Rollema C. van Roon E. Vries T. (2020). Observational study of administering intranasal steroid sprays by healthcare workers. BMJ Open 10 (8), e037660. 10.1136/bmjopen-2020-037660 32868363 PMC7462155

[B31] Djupesland P. G. C. M. J. Mahmoud R. A. (2014). The nasal approach to delivering treatment for brain diseases: an anatomic, physiologic, and delivery technology overview. Ther. Deliv. 5 (6), 709–733. 10.4155/tde.14.41 25090283

[B32] Druce H. M. Ramsey D. L. Karnati S. Carr A. N. (2018). Topical nasal decongestant oxymetazoline (0.05%) provides relief of nasal symptoms for 12 hours. Rhinology 56 (4), 343–350. 10.4193/Rhin17.150 29785414

[B33] Duzman E. Warman A. Warman R. (1986). Efficacy and safety of topical oxymetazoline in treating allergic and environmental conjunctivitis. Ann. Ophthalmol. 18 (1), 28–31. 3513687

[B34] Eccles R. Eriksson M. Garreffa S. Chen S. C. (2008). The nasal decongestant effect of xylometazoline in the common cold. Am. J. Rhinol. 22 (5), 491–496. 10.2500/ajr.2008.22.3202 18655753

[B35] Eccles R. Martensson K. Chen S. C. (2010). Effects of intranasal xylometazoline, alone or in combination with ipratropium, in patients with common cold. Curr. Med. Res. Opin. 26 (4), 889–899. 10.1185/03007991003648015 20151787

[B36] Eland I. A. Belton K. J. van Grootheest A. C. Meiners A. P. Rawlins M. D. Stricker B. H. (1999). Attitudinal survey of voluntary reporting of adverse drug reactions. Br. J. Clin. Pharmacol. 48 (4), 623–627. 10.1046/j.1365-2125.1999.00060.x 10583035 PMC2014371

[B37] Ernsberger P. Graves M. E. Graff L. M. Zakieh N. Nguyen P. Collins L. A. (1995). I1-imidazoline receptors. Definition, characterization, distribution, and transmembrane signaling. Ann. N. Y. Acad. Sci. 763, 22–42. 10.1111/j.1749-6632.1995.tb32388.x 7677333

[B38] Erszegi A. Viola R. Bahar M. A. Toth B. Fejes I. Vagvolgyi A. (2024). Not first-line antihypertensive agents, but still effective-the efficacy and safety of imidazoline receptor agonists: a network meta-analysis. Pharmacol. Res. Perspect. 12 (3), e1215. 10.1002/prp2.1215 38807350 PMC11133783

[B39] Eskiizmir G. Hirçin Z. Ozyurt B. Unlü H. (2011). A comparative analysis of the decongestive effect of oxymetazoline and xylometazoline in healthy subjects. Eur. J. Clin. Pharmacol. 67 (1), 19–23. 10.1007/s00228-010-0941-z 21069518

[B40] European Medicines Agency (2021). “EMA/164735/2021 - list of nationally authorised medicinal products,” in Active substance:oxymetazoline 2021. Available online at: https://www.ema.europa.eu/en/documents/psusa/oxymetazoline-list-nationally-authorised-medicinal-products-psusa00002258202008_en.pdf.

[B41] European Medicines Agency (2024). “EMA/156951/2024 - list of nationally authorised medicinal products,” in Active substance:xylometazoline 2024. Available online at: https://www.ema.europa.eu/en/documents/psusa/xylometazoline-list-nationally-authorised-medicinal-products-psusa-00003134-202305_en.pdf.

[B42] Evaluation of a nasal decongestant (1965). Evaluation of a nasal decongestant. Oxymetazoline hydrochloride (Afrin). JAMA 193 (13), 1115. 10.1001/jama.1965.03090130043013 4157497

[B43] Fabi M. Formigari R. Picchio F. M. (2009). Are nasal decongestants safer than rhinitis? A case of oxymetazoline-induced syncope. Cardiol. Young 19 (6), 633–634. 10.1017/S1047951109990722 19775485

[B44] Feinberg S. M. (1945). Nasal congestion from frequent use of privine hydrochloride. J. Am. Med. Assoc. 128 (15), 1095–1096. 10.1001/jama.1945.92860320001011

[B45] Gold M. S. Blum K. Bowirrat A. Pinhasov A. Bagchi D. Dennen C. A. (2024). A historical perspective on clonidine as an alpha-2A receptor agonist in the treatment of addictive behaviors: focus on opioid dependence. INNOSC Theranostics Pharmacol. Sci. 7 (3), 1918. 10.36922/itps.1918 39119149 PMC11308626

[B46] Graf P. Juto J. E. (1995). Sustained use of xylometazoline nasal spray shortens the decongestive response and induces rebound swelling. Rhinology 33 (1), 14–17. 10.4193/Rhin 7540314

[B47] Graf P. Enerdal J. Hallen H. (1999). Ten days' use of oxymetazoline nasal spray with or without benzalkonium chloride in patients with vasomotor rhinitis. Arch. Otolaryngol. Head. Neck Surg. 125 (10), 1128–1132. 10.1001/archotol.125.10.1128 10522506

[B48] Graf C. Bernkop-Schnurch A. Egyed A. Koller C. Prieschl-Grassauer E. Morokutti-Kurz M. (2018). Development of a nasal spray containing xylometazoline hydrochloride and iota-carrageenan for the symptomatic relief of nasal congestion caused by rhinitis and sinusitis. Int. J. Gen. Med. 11, 275–283. 10.2147/IJGM.S167123 30013382 PMC6037157

[B49] Greene R. R. (2005). Clinical images: Afrin-induced central nervous system vasospasm and thunderclap headache. Arthritis Rheum. 52 (10), 3314. 10.1002/art.21292 16200605

[B50] Greenstein N. M. (1955). Reactions following use of nasal decongestants. J. Am. Med. Assoc. 157 (13), 1153. 10.1001/jama.1955.02950300081021

[B51] Grimaldi-Bensouda L. Begaud B. Benichou J. Nordon C. Dialla O. Morisot N. (2021). Decongestant use and the risk of myocardial infarction and stroke: a case-crossover study. Sci. Rep. 11 (1), 4160. 10.1038/s41598-021-83718-8 33603081 PMC7893034

[B52] Gussow L. (2020). Toxicology rounds. Emerg. Med. News 42 (4), 8. 10.1097/01.Eem.0000660452.24088.14

[B53] Haenisch B. Walstab J. Herberhold S. Bootz F. Tschaikin M. Ramseger R. (2010). Alpha-adrenoceptor agonistic activity of oxymetazoline and xylometazoline. Fundam. Clin. Pharmacol. 24 (6), 729–739. 10.1111/j.1472-8206.2009.00805.x 20030735

[B54] Hagen M. Varbiro G. Montanari E. Ballerini Fernandes M. (2025). Revisiting rhinitis medicamentosa: examining the evidence on topical nasal decongestants. J. Pharm. Pract. 0 (0), 08971900251350510. 10.1177/08971900251350510 40580050

[B55] Hamdan A. T. Cherobin G. B. Voegels R. L. Rhee J. S. Garcia G. J. M (2024). Effects of mucosal decongestion on nasal aerodynamics: a pilot Study. Otolaryngology–Head Neck Surg. 170(6):1696–1704. 10.1002/ohn.713 38461407 PMC11441408

[B56] Head G. A. Mayorov D. N. (2006). Imidazoline receptors, novel agents and therapeutic potential. Cardiovasc Hematol. Agents Med. Chem. 4 (1), 17–32. 10.2174/187152506775268758 16529547

[B57] Hieble J. P. Ruffolo R. R. (1992). Imidazoline receptors: historical perspective. Fundam. Clin. Pharmacol. 6, 7S–13S. 10.1111/j.1472-8206.1992.tb00136.x 1505887

[B58] Hochban W. Althoff H. Ziegler A. (1999). Nasal decongestion with imidazoline derivatives: acoustic rhinometry measurements. Eur. J. Clin. Pharmacol. 55 (1), 7–12. 10.1007/s002280050585 10206078

[B59] Horie K. Obika K. Foglar R. Tsujimoto G. (1995). Selectivity of the imidazoline alpha-adrenoceptor agonists (oxymetazoline and cirazoline) for human cloned alpha 1-adrenoceptor subtypes. Br. J. Pharmacol. 116 (1), 1611–1618. 10.1111/j.1476-5381.1995.tb16381.x 8564227 PMC1908909

[B60] Hotovy R. Enenkel H. J. Gillissen J. Jahn U. Kieser H. Kraft H. G. (1961). On the pharmacology of 2-4-tert-butyl-2,6-dimethyl-3-hydroxybenzyl-2-imidazolinium chloride. Arzneimittelforschung 11, 1016–1022. 14449214

[B61] Hutcheon D. E. P'An S. Y. Gardocki J. F. Jaeger D. A. (1955). The sympathomimetic and other pharmacological properties of DL 2-(1,2,3,4-tetrahydro-1-naphthyl)-imidazoline (tetrahydrozoline). J. Pharmacol. Exp. Ther. 113 (3), 341–352. 10.1016/S0022-3565(25)11519-X 14368502

[B62] Jagade M. V. Langade D. G. Pophale R. R. Prabhu A. (2008). Oxymetazoline plus dexpanthenol in nasal congestion. Indian J. Otolaryngology Head and Neck Surg. 60 (4), 393–397. 10.1007/s12070-008-0125-7 23120592 PMC3476802

[B63] Jones R. S. (2021). Conceptual model for using imidazoline derivative solutions in pulpal management. J. Clin. Med. 10 (6), 1212. 10.3390/jcm10061212 33803990 PMC7998280

[B64] Kabi A. K. Gujjarappa R. Singh V. Malakar C. C. (2024). Biological impacts of imidazoline derivatives. Chem. Pap. 78 (10), 5743–5752. 10.1007/s11696-024-03496-1

[B65] Katz R. I. Hovagim A. R. Finkelstein H. S. Grinberg Y. Boccio R. V. Poppers P. J. (1990). A comparison of cocaine, lidocaine with epinephrine, and oxymetazoline for prevention of epistaxis on nasotracheal intubation. J. Clin. Anesth. 2 (1), 16–20. 10.1016/0952-8180(90)90043-3 2310576

[B66] Khan Z. P. Ferguson C. N. Jones R. M. (1999). alpha-2 and imidazoline receptor agonists. Their pharmacology and therapeutic role. Anaesthesia 54 (2), 146–165. 10.1046/j.1365-2044.1999.00659.x 10215710

[B67] Khan M. A. A. Hamid S. Babar Z.-U.-D. (2023). Pharmacovigilance in high-income countries: current developments and a review of literature. Pharmacy 11 (1), 10. 10.3390/pharmacy11010010 36649020 PMC9844306

[B68] Kishore A. Blake L. Wang C. Ba S. Gross G. (2015). Evaluating the effect of sinex® (0.05% oxymetazoline) Nasal spray on reduction of Nasal congestion using computational fluid dynamics. J. Biomech. Eng. 137 (8), 081011. 10.1115/1.4030825 26065640

[B69] Koelsch S. Tschaikin M. Sacher F. (2007). Anti-rhinovirus-specific activity of the alpha-sympathomimetic oxymetazoline. Arzneimittelforschung 57 (7), 475–482. 10.1055/s-0031-1296635 17803062

[B70] Krasavin M. (2015). Biologically active compounds based on the privileged 2-imidazoline scaffold: the world beyond adrenergic/imidazoline receptor modulators. Eur. J. Med. Chem. 97, 525–537. 10.1016/j.ejmech.2014.11.028 25466925

[B71] Kully B. M. (1945). The use and abuse of nasal vasoconstrictor medications. J. Am. Med. Assoc. 127 (6), 307–310. 10.1001/jama.1945.02860060005002

[B72] Kuzminov B. Turkina V. Кuzminov Y. (2018). Rationale for naphazoline effects in-depth study. Curr. Issues Pharm. Med. Sci. 31 (1), 29–33. 10.1515/cipms-2018-0007

[B73] Lafaurie M. Olivier P. Khouri C. Atzenhoffer M. Bihan K. Durrieu G. (2020). Myocardial infarction and ischemic stroke with vasoconstrictors used as nasal decongestant for common cold: a French pharmacovigilance survey. Eur. J. Clin. Pharmacol. 76 (4), 603–604. 10.1007/s00228-019-02807-w 31858187

[B74] Lake C. F. (1946). Rhinitis medicamentosa. Proc. Staff Meet. Mayo Clin. 21 (19), 367–371. 20274226

[B75] Langer S. Z. (1999). History and nomenclature of alpha1-adrenoceptors. Eur. Urol. 36 (Suppl. 1), 2–6. 10.1159/000052310 10393465

[B76] Leupold D. Wartenberg K. E. (2011). Xylometazoline abuse induced ischemic stroke in a young adult. Neurologist 17 (1), 41–43. 10.1097/NRL.0b013e3181d2ab04 21192193

[B77] Li J. Hu Y. Yuan Y. Zhao Y. Han Q. Liu C. (2021). Repurposing of antazoline hydrochloride as an inhibitor of hepatitis B virus DNA secretion. Virol. Sin. 36 (3), 501–509. 10.1007/s12250-020-00306-2 33165771 PMC8257819

[B78] Liu H. Du D. M. (2009). Recent advances in the synthesis of 2‐imidazolines and their applications in homogeneous catalysis. Adv. Synthesis and Catal. 351 (4), 489–519. 10.1002/adsc.200800797

[B79] Liu G. Martin W. J. Mirmozaffari Y. Ni R. Li Z. (2025). Computational modeling of nasal cavity aerodynamics: implications for surgical outcomes and targeted drug administration. Ear: Nose and Throat Journal. 10.1177/01455613251335109 PMC12928848 40257813

[B80] Ljung B. Andréasson S. (1996). Comparison of Midazolam Nasal spray to nasal drops for the sedation of children. J. Nucl. Med. Technol. 24 (1), 32–34.

[B81] Maaliki D. Jaffa A. A. Nasser S. Sahebkar A. Eid A. H. (2024). Adrenoceptor desensitization: current understanding of mechanisms. Pharmacol. Rev. 76 (3), 358–387. 10.1124/pharmrev.123.000831 38697858 PMC12164723

[B82] Malik M. Pasupuleti L. Ng C. Baroody F. (2025). A closer look at the evidence supporting appropriate use of nasal oxymetazoline HCl spray and the risk of rebound congestion: a systematic review. J. Curr. Med. Res. Opin. 8 (02), 3951–3969. 10.52845/CMRO/2025/8-2-5

[B83] Margraff F. Bertram D. (2014). Adverse drug reaction reporting by patients: an overview of fifty countries. Drug Saf. 37 (6), 409–419. 10.1007/s40264-014-0162-y 24748428

[B84] Maryadele J. O'Neil P. E. H. Koch C. B. Roman K. J. (2007). The Merck Index: an encyclopedia of chemicals, drugs, and biologicals. Merck research laboratories division of merck and Co. 14th ed. (Whitehouse Station, NJ: Inc.), 2197.

[B85] Maunsell K. (1959). The use and misuse of drugs in symptomatic treatment of the allergic nose. Postgrad. Med. J. 35 (406), 467–469. 10.1136/pgmj.35.406.467 21313640 PMC2501865

[B86] Mehedi M. S. A. Tepe J. J. (2020). Recent advances in the synthesis of imidazolines (2009–2020). Adv. Synthesis and Catal. 362 (20), 4189–4225. 10.1002/adsc.202000709

[B87] Menger H. C. (1959). New ophthalmic decongestant, tetrahydrozoline hydrochloride; clinical use in 1,156 patients with conjunctival irritation. J. Am. Med. Assoc. 170 (2), 178–179. 10.1001/jama.1959.03010020036011 13640997

[B88] Menshawey E. Menshawey R. (2024). More than meets the eye: a scoping review on the non-medical uses of THZ eye drops. Forensic Sci. Med. Pathol. 20 (2), 569–578. 10.1007/s12024-023-00680-9 37505321 PMC11297056

[B89] Mertins P. S. Jr (1947). Excessive self medication with naphazoline hydrochloride (privine hydrochloride). J. Am. Med. Assoc. 134 (14), 1175. 10.1001/jama.1947.72880310002008a 20252003

[B90] Mosges R. Shah-Hosseini K. Hucke H. P. Joisten M. J. (2017). Dexpanthenol: an overview of its contribution to symptom relief in acute rhinitis treated with decongestant nasal sprays. Adv. Ther. 34 (8), 1850–1858. 10.1007/s12325-017-0581-0 28695477 PMC5565656

[B91] Muramatsu I. Kigoshi S. (1992). Tizanidine may discriminate between imidazoline-receptors and α2-adrenoceptors. Jpn. J. Pharmacol. 59 (4), 457–459. 10.1254/jjp.59.457 1331591

[B92] Musa K. A. K. Eriksson L. A. (2007). Theoretical assessment of naphazoline redoxchemistry and photochemistry. J. Phys. Chem. B 111 (15), 3977–3981. 10.1021/jp070207f 17388561

[B93] Musgrave I. F. Hughes R. A. (1998). Investigation of I1-imidazoline receptors using microphysiometry and molecular modelling. J. Aut. Nerv. Syst. 72 (2), 137–146. 10.1016/S0165-1838(98)00098-8 9851562

[B94] Musshoff F. Madea B. Woelfle J. Vlanic D. (2014). Xylometazoline poisoning: a 40-fold nasal overdose caused by a compounding error in 3 children. Forensic Sci. Int. 238, e3–e5. 10.1016/j.forsciint.2014.02.011 24642023

[B95] Nagane Y. Utsugisawa K. Suzuki S. Masuda M. Shimizu Y. Utsumi H. (2011). Topical naphazoline in the treatment of myasthenic blepharoptosis. Muscle Nerve 44 (1), 41–44. 10.1002/mus.22002 21491460

[B96] National Center for Biotechnology Information (2024). PubChem compound summary for CID 4436, naphazoline. Available online at: https://pubchem.ncbi.nlm.nih.gov/compound/Naphazoline.

[B97] National Drug Formulary. Ministry of Health Singapore (2021). National Drug Formulary (NDF): active ingredient - oxymetazoline. Available online at: https://www.ndf.gov.sg/monograph/Detail/M00261.

[B98] Neistadt I. (1955). Clinical trial of tetrahydrozoline hydrochloride; a valuable new nasal decongestant. AMA Arch. Otolaryngol. 62 (2), 143–144. 10.1001/archotol.1955.03830020025005 14397923

[B99] Newland M. Eberly H. Ma C. Lighthall J. G. (2025). The use of oxymetazoline 0.1% ophthalmic solution for acquired blepharoptosis: a systematic review. Laryngoscope 135 (1), 8–14. 10.1002/lary.31723 39172003 PMC11635132

[B100] Nordt S. P. Vivero L. E. Cantrell F. L. (2016). Not just a drop in the bucket-inversion of oxymetazoline nasal decongestant container increases potential for severe pediatric poisoning. J. Pediatr. 168, 240–241. 10.1016/j.jpeds.2015.09.067 26522979

[B101] Norwegian Institute of Public Health (2024). WHO collaborating centre for drug statistics methodology. ATC/DDD index. Available online at: https://atcddd.fhi.no/atc_ddd_index/?code=R01AA&showdescription=yes.

[B102] Nutt D. J. French N. Handley S. Hudson A. Husbands S. Jackson H. (1995). Functional studies of specific imidazoline-2 receptor ligands. Ann. N. Y. Acad. Sci. 763 (1), 125–139. 10.1111/j.1749-6632.1995.tb32397.x 7677321

[B103] Oishi T. Kashiura M. Yasuda H. Kishihara Y. Tominaga K. Tamura H. (2024). Naphazoline intoxication managed with minimally invasive cardiac output monitoring. Am. J. Emerg. Med. 77, 233.e5–233.e7. 10.1016/j.ajem.2023.12.034 38155033

[B104] Okoro R. N. (2022). Can your patient read this? The need to move to patient-centered medication product labels and patient information leaflets. J. Am. Pharm. Assoc. 62 (5), 1528–1530. 10.1016/j.japh.2022.05.016 35672207

[B105] Ottaviano G. Fokkens W. J. (2016). Measurements of nasal airflow and patency: a critical review with emphasis on the use of peak nasal inspiratory flow in daily practice. Allergy 71 (2), 162–174. 10.1111/all.12778 26447365

[B106] Pasini C. Massara G. (1958). Rhinopathy medicamentosa in rhinological practice; clinical evaluation of a recent vasoconstrictor: tetrahydrozoline. Minerva Otorinolaringol. 8 (3), 69–72. 13541125

[B107] Passali D. Salerni L. Passali G. C. Passali F. M. Bellussi L. (2006). Nasal decongestants in the treatment of chronic nasal obstruction: efficacy and safety of use. Expert Opin. Drug Saf. 5 (6), 783–790. 10.1517/14740338.5.6.783 17044805

[B108] Patel J. Patel I. Desai D. Desai S. (2021). Ischemic stroke associated with chronic xylometazoline nasal spray misuse: a rare avoidable adverse event. Ann. Indian Acad. Neurol. 24 (2), 304–307. 10.4103/aian.AIAN_291_20 34220102 PMC8232514

[B109] Patil N. Jain S. (2024). Rhinomanometry: a comprehensive review of its applications and advancements in rhinology practice. Cureus 16 (5), e61370. 10.7759/cureus.61370 38947630 PMC11214531

[B110] Perez D. M. (2007). Structure-function of alpha1-adrenergic receptors. Biochem. Pharmacol. 73 (8), 1051–1062. 10.1016/j.bcp.2006.09.010 17052695 PMC2034198

[B111] Pigini M. Quaglia W. Gentili F. Marucci G. Cantalamessa F. Franchini S. (2000). Structure–activity relationship at α-adrenergic receptors within a series of imidazoline analogues of cirazoline. Bioorg. and Med. Chem. 8 (5), 883–888. 10.1016/S0968-0896(00)00030-4 10882000

[B112] Piletz J. E. Ordway G. A. Zhu H. Duncan B. J. Halaris A. (2000). Autoradiographic comparison of [3H]-clonidine binding to non-adrenergic sites and alpha(2)-adrenergic receptors in human brain. Neuropsychopharmacology 23 (6), 697–708. 10.1016/S0893-133X(00)00166-4 11063925

[B113] Ponomarev D. B. Remizov D. V. Kondakov A. Y. Drachyov I. S. Tikhomirov P. V. Kudryashov V. S. (2023). Experimental Study of the effectiveness of naphazoline and Co-Administration of filgrastim in combined radiation injury. Biol. Bull. 50 (11), 3054–3060. 10.1134/S1062359023110183

[B114] Pritchard S. Glover M. Guthrie G. Brum J. Ramsey D. Kappler G. (2014). Effectiveness of 0.05% oxymetazoline (Vicks Sinex micromist®) nasal spray in the treatment of objective nasal congestion demonstrated to 12 h post-administration by magnetic resonance imaging. Pulm. Pharmacol. Ther. 27 (1), 121–126. 10.1016/j.pupt.2013.08.002 23988443

[B115] Proudman R. G. W. Baker J. G. (2021). The selectivity of α-adrenoceptor agonists for the human α1A, α1B, and α1D-adrenoceptors. Pharmacol. Res. and Perspect. 9 (4), e00799. 10.1002/prp2.799 34355529 PMC8343220

[B116] Prouillac C. Célariès B. Vicendo P. Rima G. (2006). Evaluation, *in vitro*, of the radioprotection of DNA from gamma-rays by naphazoline. C R. Biol. 329 (3), 196–199. 10.1016/j.crvi.2006.01.002 16545761

[B117] Putnam L. E. Herwick R. P. (1946). Privine dependence of two years' duration. J. Am. Med. Assoc. 130 (11), 702. 10.1001/jama.1946.02870110026008 21016281

[B118] Ramey J. T. Bailen E. Lockey R. F. (2006). Rhinitis medicamentosa. J. Investig. Allergol. Clin. Immunol. 16 (3), 148–155. 16784007

[B119] Ravindra G. K. Subramaniam B. Veeranna B. (2024). Standards of child resistant packaging: a regulatory view. Ind. J. Pharm. Edu Res. 58(4):1045–4. 10.5530/ijper.58.4.116

[B120] Regunathan S. Reis D. J. (1996). Imidazoline receptors and their endogenous ligands. Annu. Rev. Pharmacol. Toxicol. 36, 511–544. 10.1146/annurev.pa.36.040196.002455 8725400

[B121] Reinecke S. Tschaikin M. (2005). Investigation of the effect of oxymetazoline on the duration of rhinitis. results of a placebo-controlled double-blind study in patients with acute rhinitis. MMW Fortschr Med. 147 (Suppl. 3), 113–118. 16261947

[B122] Rizvic E. Jankovic G. Kostic-Rajacic S. Savic M. M. (2017). Atypical sympathomimetic drug lerimazoline mediates contractile effects in rat aorta predominantly by 5-HT2A receptors. Bosn. J. Basic Med. Sci. 17 (3), 194–202. 10.17305/bjbms.2017.2071 28628756 PMC5581967

[B123] Rollema C. van Roon E. N. de Vries T. W. (2019). Inadequate quality of administration of intranasal corticosteroid sprays. J. Asthma Allergy 12, 91–94. 10.2147/JAA.S189523 31040706 PMC6452790

[B124] Ruffolo Jr. R. R. Waddell J. E. (1982). Receptor interactions of imidazolines: α-adrenoceptors of rat and rabbit aortae differentiated by relative potencies, affinities and efficacies of imidazoline agonists. Br. J. Pharmacol. 77 (1), 169–176. 10.1111/j.1476-5381.1982.tb09283.x 6289955 PMC2044657

[B125] Runkle K. (2016). Decongestants, antihistamines and nasal irrigation for acute sinusitis in children. Paediatr. Child. Health 21 (3), 143–144. 10.1093/pch/21.3.143 27398054 PMC4933076

[B126] Russo E. Giombi F. Paoletti G. Heffler E. Canonica G. W. Pirola F. (2023). Use, abuse, and misuse of nasal medications: Real-life survey on community pharmacist's perceptions. J. Pers. Med. 13 (4), 579. 10.3390/jpm13040579 37108966 PMC10142332

[B127] Samson C. R. Danzig M. R. Sasovetz D. Thompson H. S. (1980). Safety and toleration of oxymetazoline ophthalmic solution. Pharmatherapeutica 2 (6), 347–352. 7433475

[B128] Sanders J. Miller D. D. Patil P. N. (1975). Alpha adrenergic and histaminergic effects of tolazoline-like imidazolines. J. Pharmacol. Exp. Ther. 195 (2), 362–371. 10.1016/S0022-3565(25)30349-6 241844

[B129] Scadding G. (2008). Optimal management of nasal congestion caused by allergic rhinitis in children: safety and efficacy of medical treatments. Paediatr. Drugs 10 (3), 151–162. 10.2165/00148581-200810030-00004 18454568

[B130] Schafer S. G. Christen M. O. Ernsberger P. R. (1992). The second generation of centrally acting drugs: moxonidine. J. Cardiovasc. Pharmacol. 20, 7–8. 10.1097/00005344-199220004-00001 1383633

[B131] Schubbe D. Scalia P. Yen R. W. Saunders C. H. Cohen S. Elwyn G. (2020). Using pictures to convey health information: a systematic review and meta-analysis of the effects on patient and consumer health behaviors and outcomes. Patient Educ. Couns. 103 (10), 1935–1960. 10.1016/j.pec.2020.04.010 32466864

[B132] Shanler S. D. Ondo A. L. (2007). Successful treatment of the erythema and flushing of rosacea using a topically applied selective alpha1-adrenergic receptor agonist, oxymetazoline. Arch. Dermatol 143 (11), 1369–1371. 10.1001/archderm.143.11.1369 18025359

[B133] Shellans S. Rich L. F. Louiselle I. (1989). Conjunctival goblet cell response to vasoconstrictor use. J. Ocul. Pharmacol. 5 (3), 217–220. 10.1089/jop.1989.5.217 2625617

[B134] Sicard R. M. Frank-Ito D. O. (2024). Parameter characteristics in intranasal drug delivery: a key to targeting medications to the olfactory airspace. Clin. Biomech. (Bristol). 114, 106231. 10.1016/j.clinbiomech.2024.106231 38507865

[B135] Sienkiewicz K. Burzyńska M. Rydlewska-Liszkowska I. Sienkiewicz J. Gaszyńska E. (2021). The importance of direct patient reporting of adverse drug reactions in the safety monitoring process. Int. J. Environ. Res. Public Health 19 (1), 413. 10.3390/ijerph19010413 35010673 PMC8745009

[B136] Slavin R. G. (2009). Treating rhinitis in the older population: special considerations. Allergy Asthma Clin. Immunol. 5 (1), 9. 10.1186/1710-1492-5-9 20016692 PMC2794852

[B137] Sosa I. (2024). Ingestion of fluids of the ocular surface containing eye drops of imidazole derivatives-alpha adrenergic receptor agonists as paragons. Pharm. (Basel) 17 (6), 758. 10.3390/ph17060758 38931425 PMC11206365

[B138] Spiller H. Griffith J. (2008). Prolonged cardiovascular effects after unintentional ingestion of tetrahydrozoline. Clin. Toxicol. (Phila). 46 (2), 171–172. 10.1080/15563650701258070 18259967

[B139] Springer J. Pejska M. Homenda W. Zdrojewski T. Danilowicz-Szymanowicz L. Kozlowski D. (2024). Effectiveness of antazoline *versus* amiodarone, flecainide and propafenone in restoring sinus rhythm at the emergency department. Adv. Med. Sci. 69 (2), 248–255. 10.1016/j.advms.2024.04.003 38649031

[B140] Stähle H. (2000). A historical perspective: development of clonidine. Best Pract. and Res. Clin. Anaesthesiol. 14 (2), 237–246. 10.1053/bean.2000.0079

[B141] Steinberg H. (1953). A new vasoconstrictor, a preliminary report. Calif. Med. 78 (6), 507. 13059628 PMC1521764

[B142] Szabo B. (2002). Imidazoline antihypertensive drugs: a critical review on their mechanism of action. Pharmacol. and Ther. 93 (1), 1–35. 10.1016/S0163-7258(01)00170-X 11916539

[B143] Thorat S. R. (2016). Formulation and product development of Nasal spray: an overview. Sch. J. App Med. Sci. 4 (8D), 2976–2985. 10.36347/sjams.2016.v04i08.048

[B144] Tong X. Dong J. Shang Y. Inthavong K. Tu J. (2016). Effects of nasal drug delivery device and its orientation on sprayed particle deposition in a realistic human nasal cavity. Comput. Biol. Med. 77, 40–48. 10.1016/j.compbiomed.2016.08.002 27509293

[B145] Trinh L. N. Smith D. K. McCoul E. D. (2024). Redundancy of pharmacologic ingredients in over-the-counter nasal sprays. OTO Open 8 (4), e70022. 10.1002/oto2.70022 39354953 PMC11442747

[B146] Tyagi R. Tyagi V. K. Pandey S. K. (2007). Imidazoline and its derivatives: an overview. J. Oleo Sci. 56 (5), 211–222. 10.5650/jos.56.211 17898484

[B147] Uehara H. Taguchi D. Osanai T. Oe Y. Yoshimura T. Yashiro S. (2024). Naphazoline intoxication with transient QT prolongation and acute myocardial injury. J. Cardiol. Cases 29 (1), 11–14. 10.1016/j.jccase.2023.09.004 38188313 PMC10770086

[B148] Uppsala Monitoring Centre (2025). The global medicines safety database. Available online at: https://who-umc.org/vigibase-data-access/about-vigibase/.

[B149] Vaidyanathan S. Williamson P. Clearie K. Khan F. Lipworth B. (2010). Fluticasone reverses oxymetazoline-induced tachyphylaxis of response and rebound congestion. Am. J. Respir. Crit. Care Med. 182 (1), 19–24. 10.1164/rccm.200911-1701OC 20203244

[B150] Valinciute-Jankauskiene A. Kubiliene L. (2021). Adverse drug reaction reporting by patients in 12 European countries. Int. J. Environ. Res. Public Health 18 (4), 1507. 10.3390/ijerph18041507 33562536 PMC7915689

[B151] van Stralen K. J. van Tol J. E. de Wildt S. N. Becker M. L. van Houten M. A. (2023). Use of xylometazoline in hospitalised infants: is it safe? A retrospective cohort study. Arch. Dis. Child. 108 (1), 62–66. 10.1136/archdischild-2022-324127 36171065

[B152] van Velzen A. G. van Riel A. J. Hunault C. van Riemsdijk T. E. de Vries I. Meulenbelt J. (2007). A case series of xylometazoline overdose in children. Clin. Toxicol. (Phila) 45 (3), 290–294. 10.1080/15563650601033326 17453884

[B153] van Zwieten P. A. (1980). Pharmacology of centrally acting hypotensive drugs. Br. J. Clin. Pharmacol. 10, 13S–20S. 10.1111/j.1365-2125.1980.tb04899.x 6104975 PMC1430104

[B154] Vanezis P. Toseland P. A. (1980). Xylometazoline poisoning--report of a case. Med. Sci. Law 20 (1), 35–36. 10.1177/002580248002000107 6154220

[B155] Vitezić D. Rozmanić V. Franulović J. Ahel V. Matesić D. (1994). Naphazoline nasal drops intoxication in children. Arh. Hig. Rada Toksikol. 45 (1), 25–29. 8067910

[B156] Von Nordheim R. W. (1955). Naphazoline, an effective curative in the treatment of irido-cyclitis and glaucoma. Ophthalmologica 130 (1), 85–93. 10.1159/000302645 13245063

[B157] Watson S. Caster O. Rochon P. A. den Ruijter H. (2019). Reported adverse drug reactions in women and men: aggregated evidence from globally collected individual case reports during half a century. EClinicalMedicine 17, 100188. 10.1016/j.eclinm.2019.10.001 31891132 PMC6933269

[B158] Wahid N. W. B. Shermetaro C. (2023). “Rhinitis medicamentosa,” in Statpearls (Treasure Island (FL): StatPearls Publishing). Available online at: https://www.ncbi.nlm.nih.gov/books/NBK538318/. 30855902

[B159] Wang J. Mao Z. F. Cheng L. (2024). Rise and fall of decongestants in treating nasal congestion related diseases. Expert Opin. Pharmacother. 25 (14), 1943–1951. 10.1080/14656566.2024.2411009 39344778

[B160] Wenzel S. Sagowski C. Laux G. Kehrl W. Metternich F. U. (2004). Course and therapy of intoxication with imidazoline derivate naphazoline. Int. J. Pediatr. Otorhinolaryngol. 68 (7), 979–983. 10.1016/j.ijporl.2004.02.011 15183593

[B161] Westerveld G. J. Scheeren R. A. Dekker I. Griffioen D. H. Voss H. P. Bast A. (1995). Anti-oxidant actions of oxymethazoline and xylomethazoline. Eur. J. Pharmacol. 291 (1), 27–31. 10.1016/0922-4106(95)90185-x 8549644

[B162] Westerveld G. Voss H. Van der Hee R. de Haan-Koelewijn G. Den Hartog G. Scheeren R. (2000). Inhibition of nitric oxide synthase by nasal decongestants. Eur. Respir. J. 16 (3), 437–444. 10.1034/j.1399-3003.2000.016003437.x 11028657

[B163] WHO Collaborating Centre for Drug Statistics Methodology (2024). Guidelines for ATC classification and DDD assignment.

[B164] Wishart D. S. Feunang Y. D. Guo A. C. Lo E. J. Marcu A. Grant J. R. (2018). DrugBank 5.0: a major update to the DrugBank database for 2018. Nucleic Acids Res. 46 (D1), D1074–D82. 10.1093/nar/gkx1037 29126136 PMC5753335

[B165] Womack J. P. Kropa J. Jimenez Stabile M. (2018). Epistaxis: outpatient management. Am. Fam. Physician 98 (4), 240–245. 30215971

[B166] World Health Organisation (2025). Defined daily dose (DDD) - definition and general considerations. Available online at: https://www.who.int/tools/atc-ddd-toolkit/about-ddd.

[B167] World health Organisation (2024). World health Organisation. VigiAccess. Available online at: https://www.vigiaccess.org/.

[B168] Wu Y. Zeng L. Zhao S. (2021). Ligands of adrenergic receptors: a structural point of view. Biomolecules 11 (7), 936. 10.3390/biom11070936 34202543 PMC8301793

[B169] Xiao Q. Bates A. J. Cetto R. Doorly D. J. (2021). The effect of decongestion on nasal airway patency and airflow. Sci. Rep. 11 (1), 14410. 10.1038/s41598-021-93769-6 34257360 PMC8277849

[B170] Xu J. Cao S. Hubner H. Weikert D. Chen G. Lu Q. (2022). Structural insights into ligand recognition, activation, and signaling of the α _2A_ adrenergic receptor. Sci. Adv. 8 (9), eabj5347. 10.1126/sciadv.abj5347 35245122 PMC8896805

[B171] Young A. Tordoff J. Smith A. (2017). What do patients want?’ Tailoring medicines information to meet patients' needs. Res. Soc. Adm. Pharm. 13 (6), 1186–1190. 10.1016/j.sapharm.2016.10.006 27818214

[B172] Zatovkaňuková P. Slíva J. (2024). Diverse pharmacovigilance jurisdiction-the right way for global drug safety? Eur. J. Clin. Pharmacol. 80 (3), 305–315. 10.1007/s00228-023-03608-y 38135821

